# Revisiting COVID-19 vaccine hesitancy around the world using data from 23 countries in 2021

**DOI:** 10.1038/s41467-022-31441-x

**Published:** 2022-07-01

**Authors:** Jeffrey V. Lazarus, Katarzyna Wyka, Trenton M. White, Camila A. Picchio, Kenneth Rabin, Scott C. Ratzan, Jeanna Parsons Leigh, Jia Hu, Ayman El-Mohandes

**Affiliations:** 1 Barcelona Institute for Global Health (ISGlobal), Hospital Clínic, University of Barcelona, Barcelona, Spain; 2 Graduate School of Public Health & Health Policy, City University of New York (CUNY), New York, NY US; 3 School of Health Administration, Dalhousie University, Halifax, NS Canada; 4 University of Calgary, Calgary, AB Canada

**Keywords:** Public health, Communication

## Abstract

The COVID-19 pandemic continues to impact daily life, including health system operations, despite the availability of vaccines that are effective in greatly reducing the risks of death and severe disease. Misperceptions of COVID-19 vaccine safety, efficacy, risks, and mistrust in institutions responsible for vaccination campaigns have been reported as factors contributing to vaccine hesitancy. This study investigated COVID-19 vaccine hesitancy globally in June 2021. Nationally representative samples of 1,000 individuals from 23 countries were surveyed. Data were analyzed descriptively, and weighted multivariable logistic regressions were used to explore associations with vaccine hesitancy. Here, we show that more than three-fourths (75.2%) of the 23,000 respondents report vaccine acceptance, up from 71.5% one year earlier. Across all countries, vaccine hesitancy is associated with a lack of trust in COVID-19 vaccine safety and science, and skepticism about its efficacy. Vaccine hesitant respondents are also highly resistant to required proof of vaccination; 31.7%, 20%, 15%, and 14.8% approve requiring it for access to international travel, indoor activities, employment, and public schools, respectively. For ongoing COVID-19 vaccination campaigns to succeed in improving coverage going forward, substantial challenges remain to be overcome. These include increasing vaccination among those reporting lower vaccine confidence in addition to expanding vaccine access in low- and middle-income countries.

## Abstract (web summary)

Vaccine hesitancy is a public health challenge. Here the authors examine COVID-19 vaccine hesitancy in June 2021 using a survey including individuals from 23 countries, and report differences compared to a year earlier.

## Introduction

Vaccine hesitancy, defined by the World Health Organization Strategic Advisory Group for Emergencies (WHO SAGE) Working Group on Vaccine Hesitancy as the “delay in acceptance or refusal of vaccination despite availability of vaccination services^1^,” was declared by WHO in 2019 as one of the ten greatest global health threats^2^. In June 2020, with no vaccine approved and as most countries were still experiencing the initial surge of SARS-CoV-2 transmission, the authors of this study reported low COVID-19 vaccine acceptance among more than 13,000 respondents in 19 of the world’s hardest-hit countries at that time^3^. As of 14 December 2021, nearly one billion individuals globally were partially vaccinated and another 3.64 billion were fully vaccinated against COVID-19; however, more than 44% of the world, predominantly in low- and middle-income countries (LMIC), were still unvaccinated^4^ and case rates, hospitalizations, and mortality remained high globally.

Vaccination is one of the most effective interventions to control the ongoing pandemic, but COVID-19 vaccination acceptance rates vary greatly globally^5–8^. Effective and comprehensive vaccination strategies require an up-to-date understanding of the perceptions that drive COVID-19 vaccine hesitancy and the common characteristics of people who are less likely to accept a vaccine or vaccination requirement, or mandate^9^.

COVID-19 vaccine hesitancy literature focuses on four interrelated subjects: (1) vaccine safety; (2) vaccine efficacy; (3) perception of risk; and (4) mistrust of governments and health and scientific institutions^10–19^. Individually-reported beliefs regarding COVID-19 vaccine safety arise from real or hypothetical knowledge of adverse events in addition to disinformation or misinformation^10^, which are often hyperbolic, proliferate on social media, and are attributed to a well-known sub-set of political and religious leaders and self-identified medical professionals^14^. Exposure to online misinformation on COVID-19 vaccine safety and efficacy can lead to a decrease in intent to receive a COVID-19 vaccine^12^. A survey conducted in nine LMICs in Asia and Africa demonstrated an inverse association between vaccine hesitancy and perceptions of effectiveness^15^. Misperception of the severity of COVID-19 infections and an individual’s risk of contracting SARS-CoV-2 are also associated with vaccine hesitancy^15–18^, which is consistent with the general literature on vaccines and risk perception^20^. When coupled with public mistrust towards health care workers (HCWs), scientific institutions, and/or health authorities, these drivers can potentially halt progress on vaccine uptake in settings where vaccines are available^21^. Understanding the characteristics and degrees of hesitancy among those who believe misinformation on safety, efficacy, and risk can help health authorities, community leaders, and other trusted sources to identify priority groups for targeted information about the safety and efficacy of available COVID-19 vaccines, which all reduce the risks of severe illness and mortality.

Demographic and other factors associated with vaccine hesitancy include residing in a rural area, lower income, female gender, lower education, and vaccine costs^15,17,18,22,23^. Studies investigating COVID-19 vaccine hesitancy among racial and ethnic minorities indicate that acceptance has improved over time^18,24–26^. Specific drivers of hesitancy among minority racial groups include mistrust of health authorities^27^ and inequitable or under-representation in vaccine trials^10^.

This survey, undertaken in late June 2021, assesses vaccine uptake and the reasons for vaccine hesitancy among participants in 23 countries, which represent approximately 60% of the world’s population^28^. It was carried out within the context of a year of substantial but very uneven global COVID-19 vaccine availability, administration, and acceptance, which necessitated new assessments of the drivers of vaccine hesitancy and the characteristics of people not vaccinated. Additions to the 2021 study include analyses of the associations between vaccine hesitancy and each of four vaccine-specific perceptions (i.e., risk, trust, safety, and efficacy), as well as a composite score of them (COVID-VAC), and the association between mental health and vaccine hesitancy, as well as support for a range of requirements for proof of vaccination to participate in activities and travel, and vaccination for children.

## Results

### Sample characteristics

23,000 participants from 23 countries responded to the survey. Approximately half were female (50.2%) and resided in LMICs (52.2%), while three-fifths (59.9%) were aged 30–59 and one-fifth (22.4%) were university graduates (Table 1, Table 2). HCWs represented one in ten (10.8%) of all respondents. COVID-19 illness (self or family) or loss of a family member to COVID-19 were most commonly reported in Ecuador (48.3% and 19.2%), Brazil (38.3% and 18.3%), and Peru (35.5% and 34.7%), and least commonly in China (1.5% and 2%), Singapore (3.3% and 5.3%), South Korea (3.8% and 2.1%), Ghana (5.8% and 1.5%), and Nigeria (6% and 2.3%). Loss of income was highest in lower-middle and upper-middle income countries (range, 31.7% in Germany to 95.1% in Ecuador). Reported experience of anxiety rates ranged from 9.2% in Ghana to 44.7% in Turkey, while experience of depression was lowest in China (12.6%) and Ghana (12.9%) and highest in Turkey (38.7%).

**Table 1:** Sample characteristics by country (*n* = 23) and the global average.

Country	Brazil	Canada	China	Ecuador	France	Germany	Ghana	India	Italy	Kenya	Mexico	Nigeria	Peru	Poland	Russia	South Africa	South Korea	Singapore	Spain	Sweden	Turkey	United Kingdom	United States	Global average
	%	%	%	%	%	%	%	%	%	%	%	%	%	%	%	%	%	%	%	%	%	%	%	%
Age groups																								
18–29	19	16.5	16.5	23.5	16.5	14.1	35.6	24.2	18.5	37.9	23.5	31.9	23.3	16	15.8	25	18.4	20.7	15.5	15.7	22.1	16.6	17.1	21
30–39	20.3	17.7	22.4	22.1	15.2	15.3	64.4	21.2	20	20.7	22.1	68.1	21.5	17.3	17.7	23.4	21.3	20.7	17.4	16.3	20.6	17.2	15.8	23.4
40–49	19	16.5	23.5	22.1	16.5	16.5		21.2	20	18.9	20.6		20.6	18.5	19	20.3	19.9	22	18.6	17.6	20.6	17.8	17.1	19.3
50–59	24.1	16.5	16.5	14.7	17.7	17.6		16.7	16.9	13.8	16.2		16.5	17.3	18.7	17.2	18.4	17.1	17.4	15.7	17.6	16.6	18.4	17.2
60+	17.7	32.9	21.2	17.6	34.2	36.5		16.7	24.6	8.8	17.6		18	30.9	28.8	14.1	22	19.5	31.1	34.7	19.1	31.8	31.6	24.3
Sex																								
Male	49.1	49.3	51.5	49.7	49.2	48.9	50.6	50.4	48.7	49.3	49.8	50.5	49.4	48.1	46.2	48.9	49.8	49.3	48.9	49.6	48.7	49.1	48.9	49.3
Female	50.9	50.1	48.5	49.7	50.7	50.5	49.2	48	51	49.9	50.1	49.2	49.9	51.5	53.2	50.6	49.7	50.2	50.7	49.6	49.9	50.4	51	50.2
Prefer not to say/Other		0.6		0.6	0.1	0.6	0.2	1.6	0.3	0.8	0.1	0.3	0.7	0.4	0.6	0.5	0.4	0.5	0.4	0.7	1.4	0.5	0.1	0.54
Education (university degree)																								
No	82.6	73.8	88	88	82	75	85	91	85.6	97	84	91.5	84	75	45	92.5	44.9	67.8	67	75.7	79	65.6	64.3	77.6
Yes	17.4	26.2	12	12	18	25	15	9	14.4	3	16	8.5	16	25	55	7.5	55.1	32.2	33	24.3	21	34.4	35.7	38.7
Income (country median)																								
More than Median	32.1	39.8	65.9	30.3	39.6	30.2	17.7	66.6	29.8	36.1	49.6	17.9	40.7	40.2	24.4	65.6	50.4	35.4	37.6	22.6	32.3	43	42	38.7
Less than Median	58.2	53	29.3	36.7	53.2	61.1	42.8	19.4	57.8	28.2	42.4	48	38.9	53.4	69.3	18.9	37.4	54.1	52.3	68.8	56.9	49	46.1	46.7
No income	9.7	7.2	4.9	33	7.2	8.7	39.5	14	12.4	35.7	8	34.1	20.3	6.4	6.3	15.5	12.2	10.4	10	8.6	10.8	8	11.9	14.6

**Table 2:** Sample characteristics by country (*n* = 23) and the global average.

Country	Brazil	Canada	China	Ecuador	France	Germany	Ghana	India	Italy	Kenya	Mexico	Nigeria	Peru	Poland	Russia	South Africa	South Korea	Singapore	Spain	Sweden	Turkey	United Kingdom	United States	Global average
	%	%	%	%	%	%	%	%	%	%	%	%	%	%	%	%	%	%	%	%	%	%	%	%
Have you or anyone else in your household experienced a loss in income due to the COVID-19 pandemic?																								
Severe loss	29.7	16.7	14.4	58.9	14.5	13.7	43.4	57.5	14.9	59.9	33.1	56.2	37.3	10.8	24.9	40.1	19.5	23.2	16.4	13.1	38.4	11.9	17.9	29
Moderate loss	42.2	24.8	51.5	36.2	23.1	18	25.9	27.2	41.2	29.3	45.2	20.6	50	16.6	36.2	39.7	40.7	39.2	34.6	23.4	37	24.7	18.2	32.4
No	28.2	58.5	34.1	5	62.5	68.2	30.7	15.2	43.9	10.7	21.7	23.2	12.6	72.5	38.9	20.2	39.9	37.6	49.1	63.5	24.6	63.4	63.8	38.6
COVID-19 experience																								
None	43.3	84.9	96.5	32.4	68.8	85.7	92.7	70.3	73.2	78	50.4	91.7	29.8	52.2	53.1	58.5	94.1	91.4	72.5	67.4	60.1	73.6	71	69.2
Self/family member sick	38.3	11	1.5	48.3	25.4	9.4	5.8	8.7	20.1	15.2	28.2	6	35.5	41.4	42.8	25.1	3.8	3.3	19.6	27.3	31.3	17.8	17.7	21
Lost family member	18.3	4.1	2	19.2	5.8	4.9	1.5	20.9	6.7	6.8	21.4	2.3	34.7	6.4	4.1	16.5	2.1	5.3	7.8	5.3	8.6	8.6	11.3	9.8
Health care worker																								
Yes	5.5	9.8	9	7.7	10.8	12	20.6	25.4	5.6	10.9	8.9	19.4	8.2	4.7	2.9	6.7	11.2	7.7	7.8	13.8	9.3	14.8	16.5	10.8
No	94.5	90.2	91	92.3	89.2	88	79.4	74.6	94.4	89.1	91.1	80.6	91.8	95.3	97.1	93.3	88.8	92.3	92.2	86.2	90.7	85.2	83.5	89.2
Mental health																								
Anxiety	25.1	24.9	12.3	21.7	28.7	20.6	9.2	26.7	26.1	24.4	29.4	13.3	39.3	20.6	36.6	37	22.7	20.8	24.4	24.1	44.7	27.6	21.9	25.3
Depression	25.3	23.1	12.6	23.1	23.1	19.2	12.9	15	22.4	32.5	23.8	25.1	28.8	20.3	27.2	30.9	21.1	21.6	22.2	23.8	38.7	25.9	23.2	23.6

Experience of anxiety and depression was defined as having symptoms for a moderate amount of time (3–4 days per week) or most or all of the time (5–7 days per week).

### Vaccine acceptance and hesitancy, June 2021

**Fig. 1:**
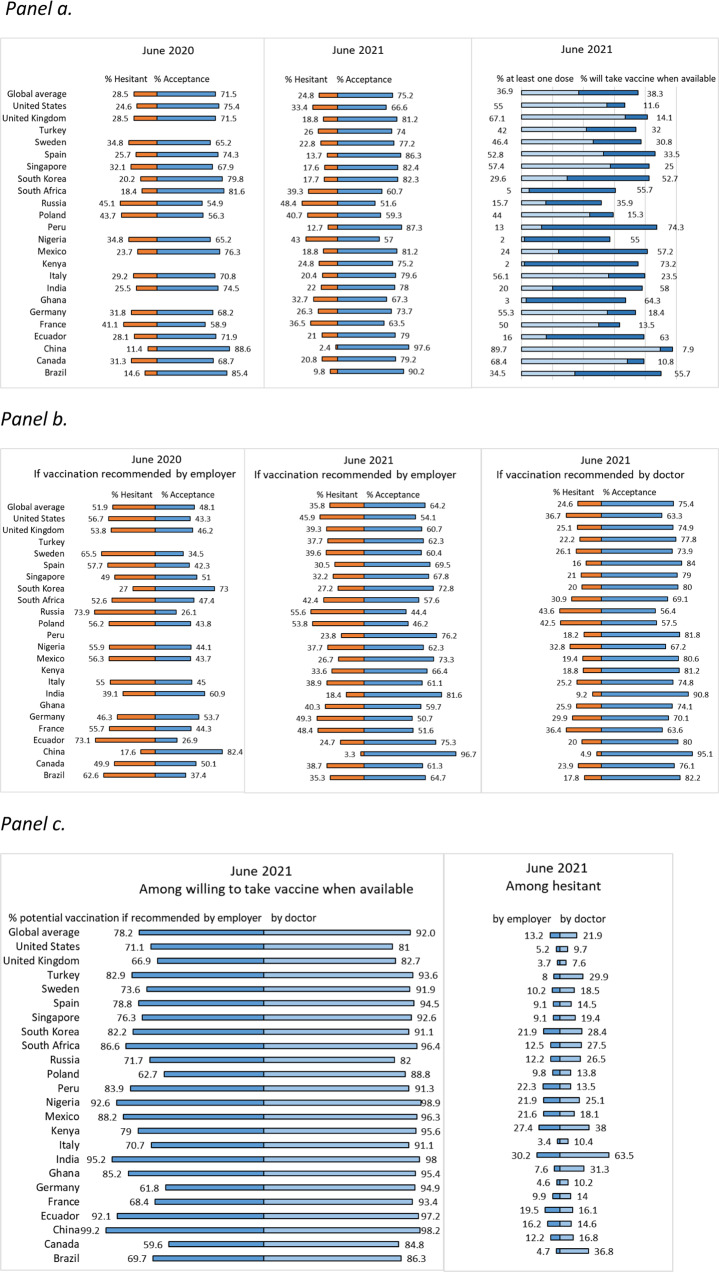
COVID-19 vaccine hesitancy and acceptance in June 2020 and June 2021. **a** COVID-19 vaccine hesitancy and acceptance. **b** COVID-19 vaccine hesitancy and acceptance if recommended by employer or one’s doctor. **a**, **b** COVID-19 acceptance in June 2020 was defined as willingness to take vaccine if proven safe and effective. COVID-19 vaccine acceptance in June 2021 was defined as having received at least one dose of a COVID-19 vaccine and if not, willingness to take the COVID-19 vaccine when it is available to them. Vaccine hesitancy was defined as having reported “no” to the question on whether they have received at least one dose of a COVID-19 vaccine and also either unsure/no opinion, somewhat disagree, or strongly disagree to the question on whether they would take a COVID-19 vaccine when available to them. Four countries (Ghana, Kenya, Peru, and Turkey) were not included in the 2020 global survey. **c** Potential COVID-19 vaccine acceptance if recommended by employer or one’s doctor among those willing to take vaccine when available and those hesitant to vaccinate. **c** Potential COVID-19 vaccination was defined as willingness to take the COVID-19 vaccine when it is available if recommended by employer or by doctor.

In June 2021, COVID-19 vaccine acceptance in the 23 countries surveyed was 75.2%, compared to 71.5% one year earlier^3^. COVID-19 vaccine acceptance at that time was defined as having received at least one dose of a COVID-19 vaccine and, if not, willingness to take the COVID-19 vaccine when it is available to them. A year earlier, in June 2020, COVID-19 acceptance was defined as willingness to get vaccinated if proven safe and effective. Of those who reported acceptance, 49% reported that they had received at least one vaccine dose and 51% said they were willing to get vaccinated once it became available to them. Vaccine hesitancy was defined as having reported “no” to the question on whether they received at least one dose of a COVID-19 vaccine and also either “unsure/no opinion,” “somewhat disagree,” or “strongly disagree” to the question on whether they would take a COVID-19 vaccine when available to them. While overall vaccine hesitancy decreased from 28.5% to 24.8%, the opposite trend was observed in South Africa (20.9% greater hesitancy), the United States (US) (8.8%), Nigeria (8.2%), and Russia (3.3%). In June 2021, vaccine hesitancy was reported most frequently in Russia (48.4%), Nigeria (43%), and Poland (40.7%) and least often in China (2.4%), the United Kingdom (UK) (18.8%), and Canada (20.8%) (Fig. 1).

The hypothetical recommendation of an employer to receive a COVID-19 vaccine was perceived more positively in all countries (a decrease in hesitancy from 52.8% in 2020 to 35.8% in 2021), except in Germany and South Korea, where 3% and 0.2%, respectively, said that they were more hesitant to accept vaccination based on an employer’s recommendation than in the previous year. In 2021, reported hesitancy associated with a hypothetical doctor’s recommendation ranged from 4.9% in China to 43.6% in Russia and was lower compared to a recommendation from one’s employer (from 3.3% in China to 55.6% in Russia). Notably, among respondents who stated that they were hesitant to vaccinate, potential vaccine acceptance was more likely to occur if recommended by one’s doctor in India (63.5%), Kenya (38%), Brazil (36.8%), Turkey (29.9%), South Korea (28.4%), and Russia (26.5%) (Fig. 1).

**Fig. 2:**
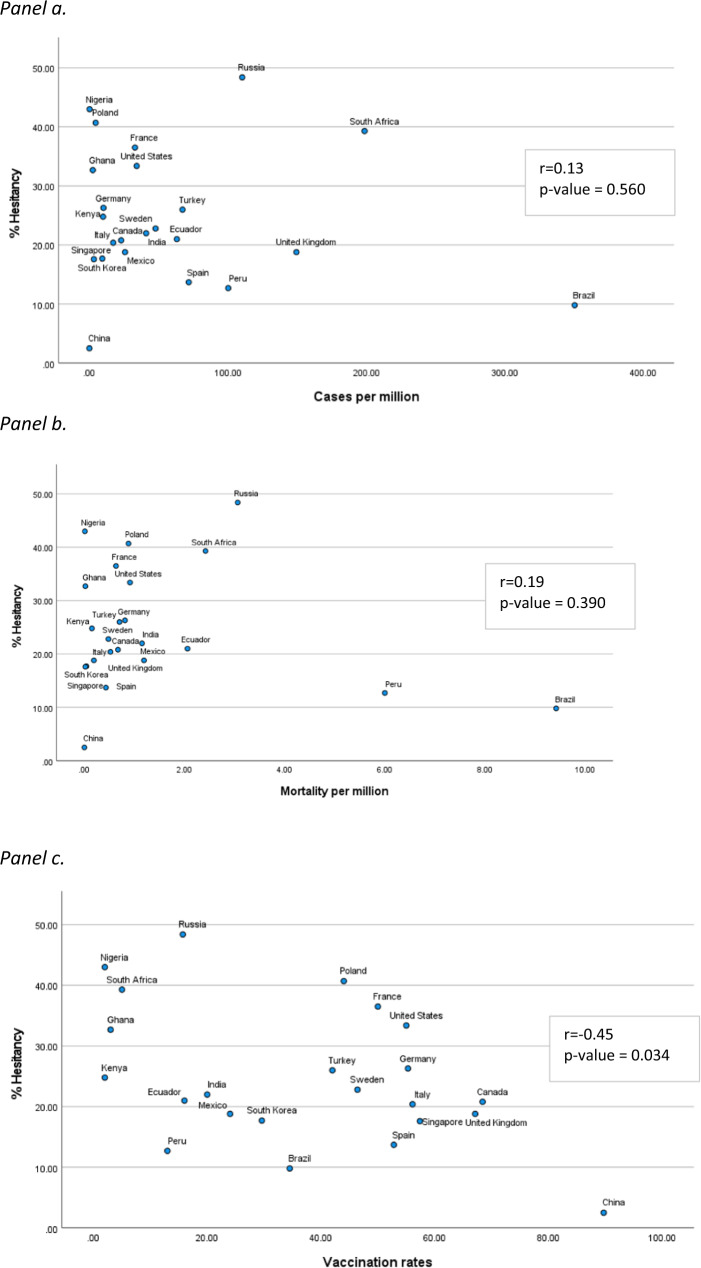
COVID-19 vaccine hesitancy by current cases and mortality. **a** COVID-19 vaccine hesitancy and COVID-19 cases. **b** COVID-19 vaccine hesitancy and COVID-19 mortality. **a**, **b** Source data are provided in a Source Data file. **c** Vaccination rates and COVID-19 vaccine hesitancy. **c** The association between a country’s COVID-19 vaccine hesitancy and COVID-19 cases and mortality (per million population) at the time of survey were each assessed using Pearson correlations and associated p-values based on two-sided tests. No adjustments for multiple analyses were made. Source data are provided in a Source Data file.

**Fig. 3:**
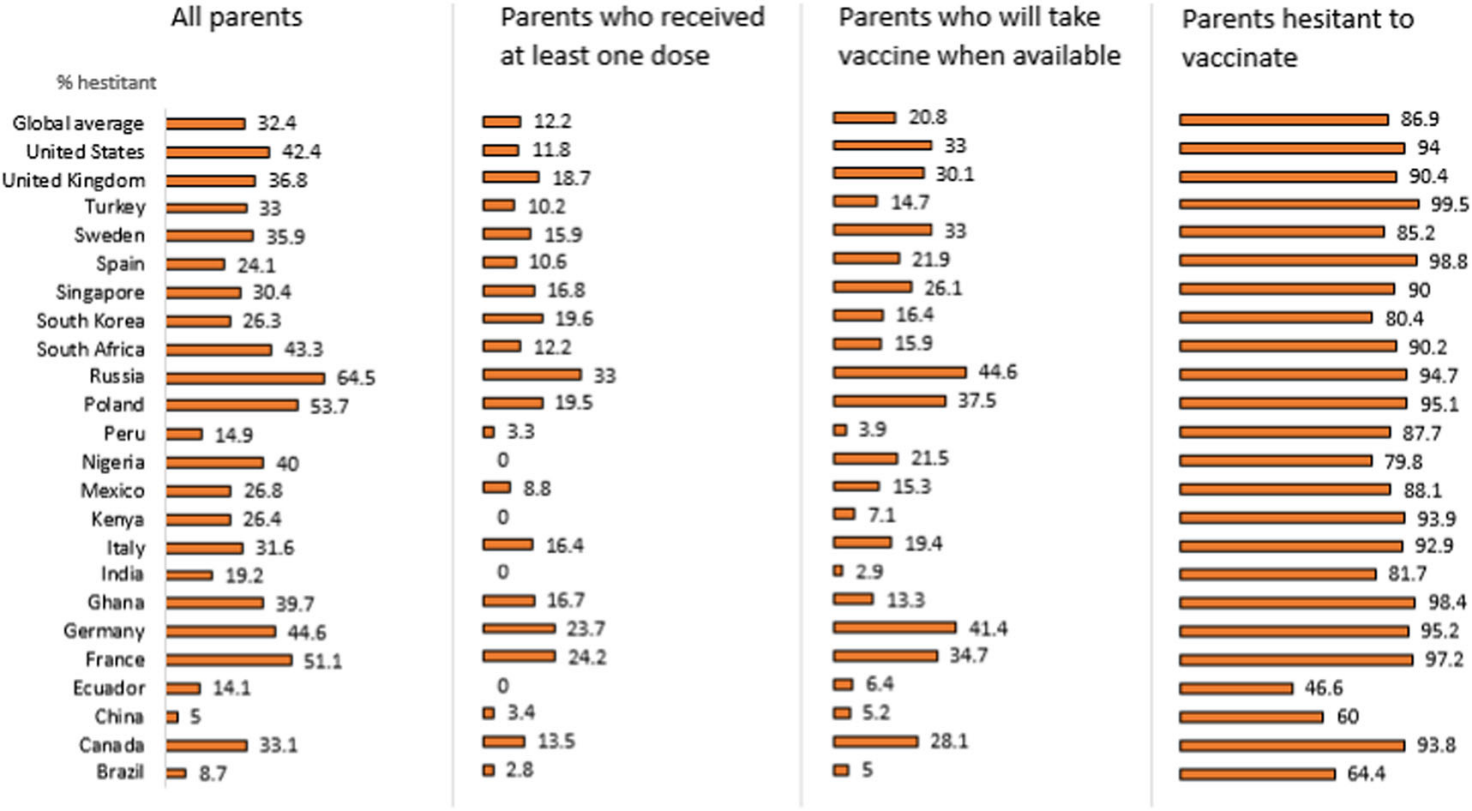
COVID-19 vaccine hesitancy for children among parents. Vaccine hesitancy was defined as having reported “no” to the question on whether respondents received at least one dose of a COVID-19 vaccine and also either “unsure/no opinion,” “somewhat disagree,” or “strongly disagree” to the question on whether they will take COVID-19 vaccine when available to them.

Vaccine hesitancy did not significantly correlate with a country’s current COVID-19 case burden (*r* = −0.13, *p* = 0.560) and mortality (*r* = −0.25, *p* = 0.390) (Fig. 2). Country vaccination rates^4^ were negatively associated with vaccine hesitancy (*r* = −0.45, *p* = 0.034) (Fig. 2). Vaccine uptake often depends on availability of supplies and services in the country. Our results found low vaccination uptake and relatively high hesitancy in countries in Africa compared with the rest of the sample. Low hesitancy was observed in countries with vaccine uptake of greater than 40% of the population, with the exception of Poland, France, and the US, which had higher hesitancy. Parents’ hesitancy to vaccinate their children was repored most often in Russia (64.5%), Poland (53.7%), and France (51.1%) and least often in China (5%), Brazil (8.7%), Ecuador (14.1%), and Peru (14.9%). In all countries, hesitancy to vaccinate one’s children was greater among parents who themselves were hesitant (Fig. 3).

### Correlates of vaccine hesitancy, June 2021

**Fig. 4:**
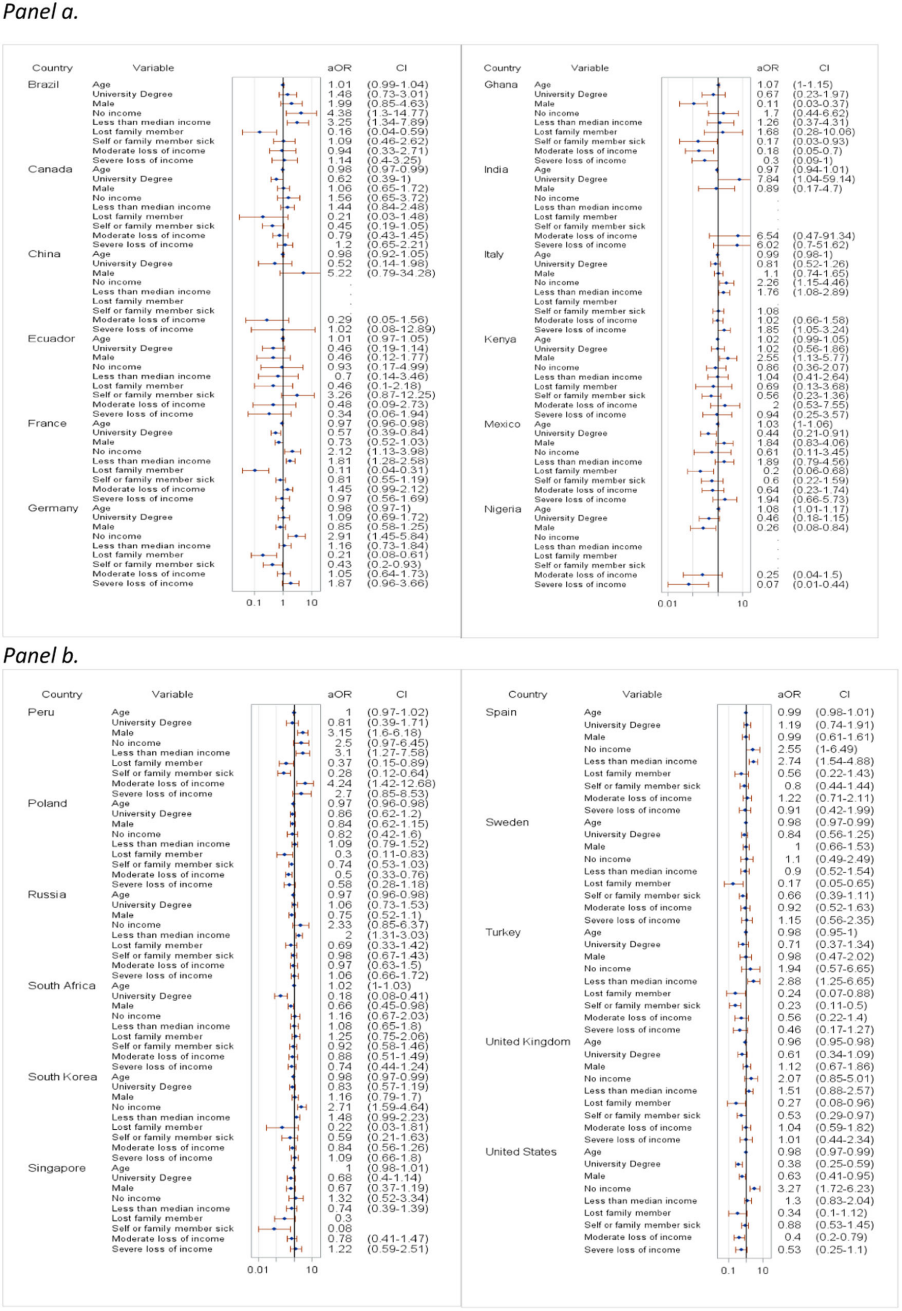
Correlates of COVID-19 vaccine hesitancy with socio-demographic factors and COVID-19 experience. **a** Correlates in Brazil, Canada, China, Ecuador, France, Germany, Ghana, India, Italy, Kenya, Mexico, and Nigeria. **b** Correlates in Peru, Poland, Russia, South Africa, South Korea, Singapore, Spain, Sweden, Turkey, the United Kingdom, and the United States. **a**, **b** Adjusted odds ratios (aOR) and 95% CI error bars (log scale) from weighted multivariable logistic regression; reference categories: Female, No university degree, More than median income, No COVID-19 sickness/death, No loss of income.

In multivariable models, vaccine hesitancy was significantly associated with younger respondents in Canada, France, Germany, South Korea, Sweden, the UK, and the US (aOR range, 0.96–0.98) (Fig. 4) and older ones in Ghana (aOR = 1.07, 95% CI [1.0, 1.15]) and Nigeria (aOR = 1.08, 95% CI [1.01, 1.17]). Male gender was significantly associated with vaccine hesitancy in Kenya and Peru (aOR = 2.55, 95% CI [1.13, 5.77] and aOR = 3.15, 95% CI [1.6, 6.18]) and female gender in Ghana, Nigeria, South Africa, and the US (aOR range, 0.11–0.66). Having a university degree was significantly negatively associated with vaccine hesitancy in France, Mexico, South Africa, and the US (aOR range, 0.18–0.57). Personal or family COVID-19 illness were significantly negatively associated with vaccine hesitancy in Germany, Ghana, Peru, Poland, Turkey, and the UK (aOR range, 0.17–0.74) and loss of a family member due to COVID-19 was significantly negatively associated with vaccine hesitancy in Brazil, France, Germany, Mexico, Peru, Poland, Singapore, Sweden, Turkey, and the UK (aOR range, 0.11–0.37). Having little or no income was significantly associated with vaccine hesitancy in Brazil, France, Germany, Italy, Nigeria, Peru, Russia, South Korea, Spain, Turkey, and the US (aOR range, 1.76–4.38). Moderate or severe income loss due to the pandemic was significantly negatively associated with vaccine hesitancy in Ghana, Nigeria, Poland, and the US (aOR range, 0.07–0.50).

**Fig. 5:**
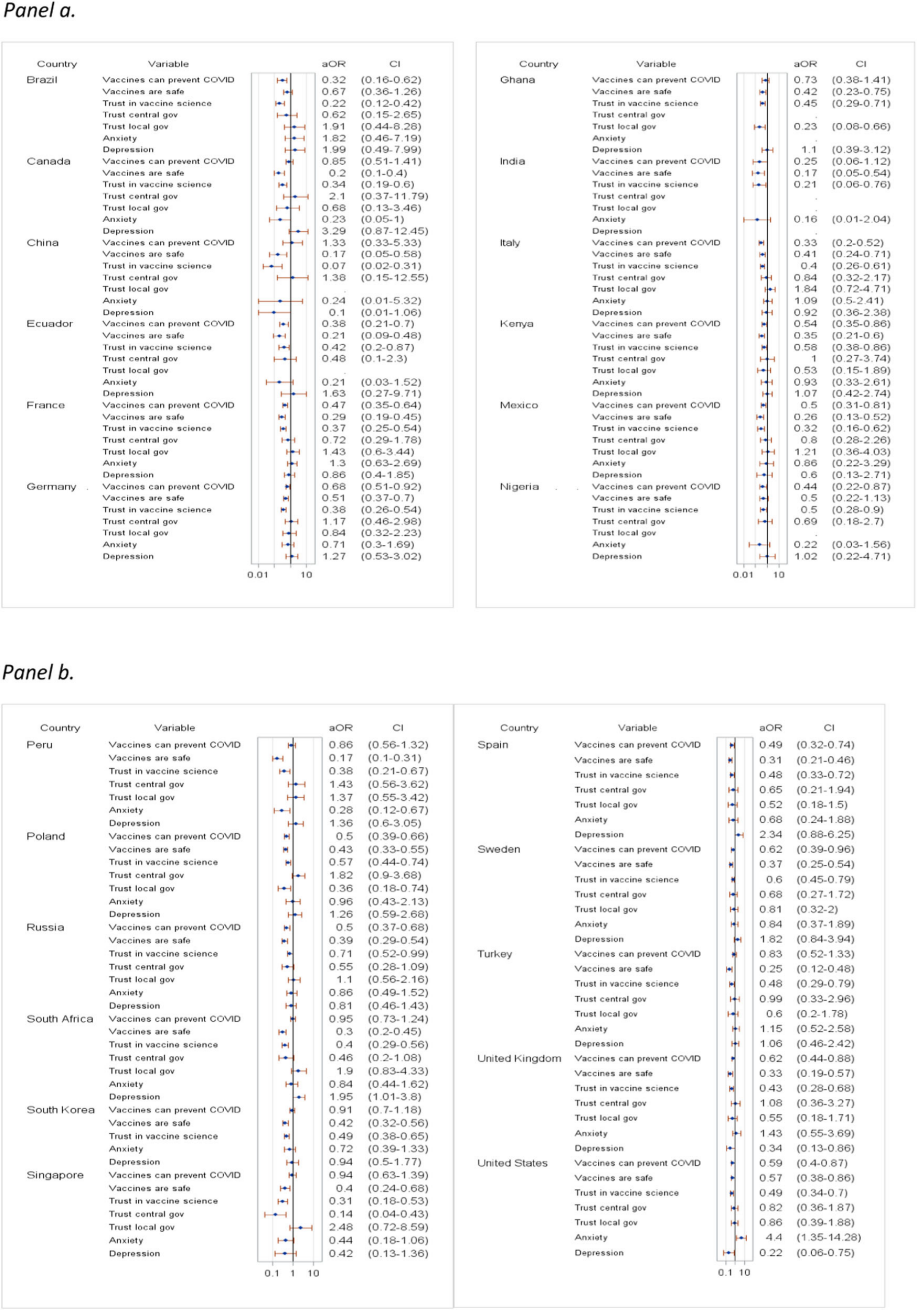
Correlates of COVID-19 vaccine hesitancy with beliefs in a vaccine’s ability to prevent COVID-19, safety and trust in the vaccine science, trust in government, anxiety, and depression. **a** Correlates in Brazil, Canada, China, Ecuador, France, Germany, Ghana, India, Italy, Kenya, Mexico, and Nigeria. **b** Correlates in Peru, Poland, Russia, South Africa, South Korea, Singapore, Spain, Sweden, Turkey, the United Kingdom, and the United States. **a**, **b** Adjusted odds ratios (aOR) and 95% CI error bars (log scale) from weighted multivariable logistic regression, adjusted for socio-demographic factors and COVID-19 experience.

In univariable analyses, vaccine hesitancy significantly correlated with negative perceptions of risk, trust, safety, and efficacy (Supplementary Table 1). The strongest correlations were observed with the statements: “I trust the science behind the COVID-19 vaccines,” “The COVID-19 vaccines available to me are safe,” and “COVID-19 can be prevented by vaccination.” Adjusted for socio-demographic factors and COVID-19 experience, these three statements were the most salient and consistent negative correlates of vaccine hesitancy in all countries (aOR range, 0.07–0.73) (Fig. 5). As a composite score (averaged scores for the six items assessing perceptions of risk, trust, safety, and efficacy), COVID-VAC was correlated with vaccine acceptance (*r* = 0.85, *p* < 0.001) and had high internal reliability (Cronbach’s alpha of 0.86) (Supplementary Table 1, Supplementary Figure 1). In univariable analyses, vaccine hesitancy was significantly lower among respondents who reported that they trust their central and/or local government in all countries except Brazil, China, Ecuador, and Peru (Supplementary Table 2). After adjustment for socio-demographic factors and COVID-19 experience, vaccine hesitancy was significantly negatively associated with trust in the central government in Singapore (aOR = 0.14, 95% CI [0.04, 0.43]) and trust in the local government in Ghana (aOR = 0.23, 95% CI [0.08, 0.66]) and Poland (aOR = 0.36, 95% CI [0.18, 0.74]).

Finally, vaccine hesitancy was significantly negatively correlated with the experience of anxiety in Canada (aOR = 0.23, 95% CI [0.05, 1]) and Peru (aOR = 0.28, 95% CI [0.12, 0.67]) but positively correlated with the experience of anxiety in South Korea (aOR = 2.42, 95% CI [1.36, 4.32]) and the US (aOR = 4.40, 95% CI [1.35, 14.28]). Experience of depression was a significant positive correlate of vaccine hesitancy in South Africa (aOR = 1.95, 95% CI [1.01, 3.80]) but a negative one in the UK (aOR = 0.34, 95% CI [0.13, 0.86]) and the US (aOR = 0.22, 95% CI [0.06, 0.75]) (Fig. 5).

### Hesitancy among health care workers (HCWs), 2021

Vaccine hesitancy was significantly lower among HCWs globally compared to non-HCWs (8.1% vs. 17%, *p* < 0.001, aOR = 0.58, 95% CI [0.47, 0.72]) (Table 3). Vaccine hesitancy was lowest among physicians (3.1%), followed by nurses (6.5%), community health workers (7.8%), and other HCWs (14.0%).

**Table 3:** COVID-19 vaccine hesitancy and acceptance by health care worker status.

		At least one dose received	Will take vaccine when available	Vaccine acceptance	Vaccine hesitancy		
	*N*	%	%	%	%	*p* value	aOR (95% CI)
Not HCW	19840	52	31	83	17		
All HCWs	3295	72.4	19.4	91.8	8.1	<0.001	0.58 (0.47, 0.72)
Physician	891	85.6^a^	11.2	96.9	3.1		
Nurse	619	74.5	19.1	93.5	6.5		
Community health worker	790	69.6	22.5	92.2	7.8		
Other health care worker	995	61.6	24.5	86.1	13.9		

Different subscripts denote statistically significant pairwise differences.

^a^OR, 95% CI and *p* value (two-sided) are from multivariable logistic model (outcome: vaccine hesitancy) after adjusting for demographic variables, COVID-19 experience, and clustering of health care workers (HCWs) in countries.

### Proof of vaccination mandates

**Fig. 6:**
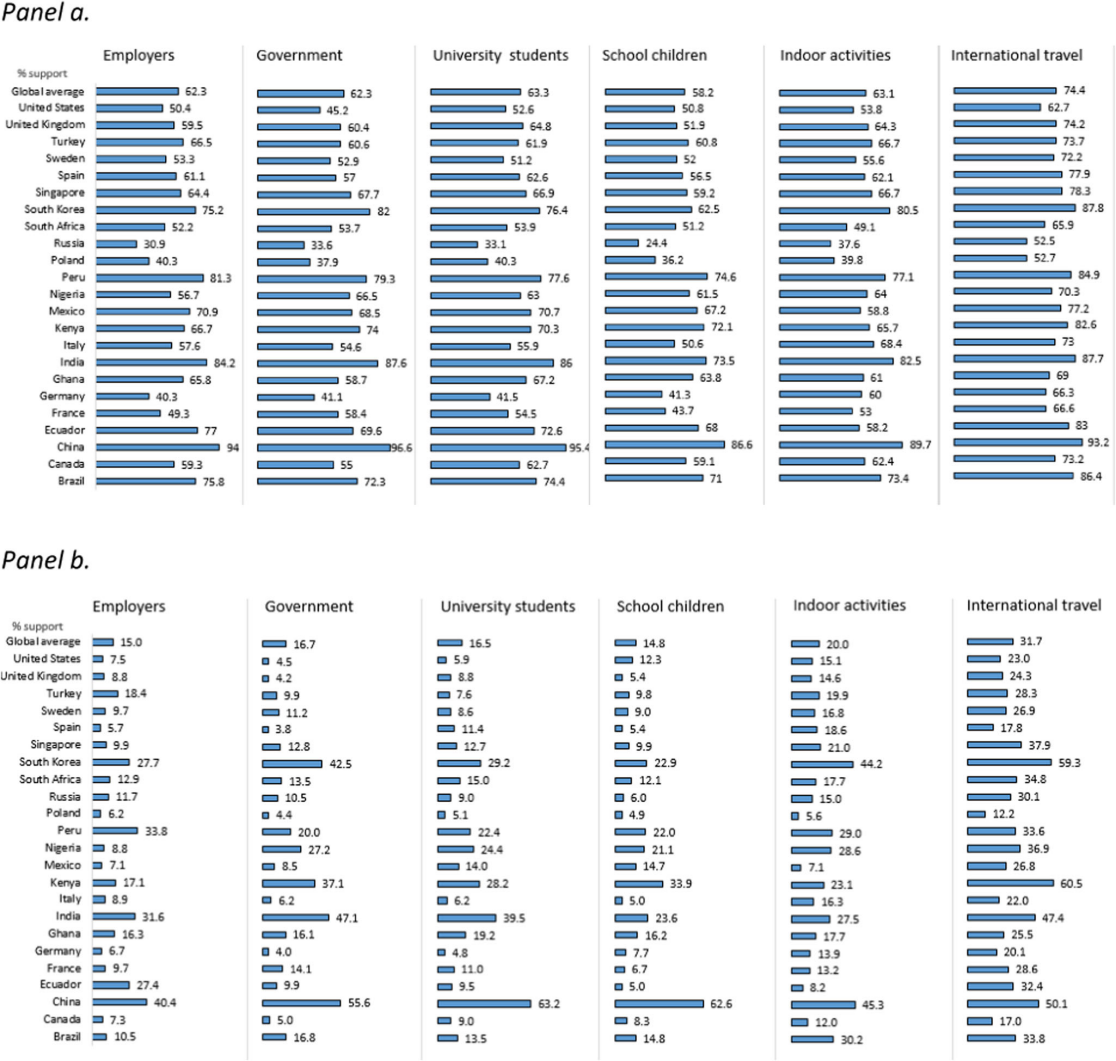
Support for COVID-19 vaccination mandates. **a** Overall. **b** Among those hesitant to vaccinate against COVID-19. **a**, **b** Support is defined as reporting “strongly agree/agree” with each mandate.

Overall, among the countries sampled, support for requiring vaccines was highest in China, India, Peru, and South Korea and lowest in Poland, Russia, and Germany. Strong support was reported globally (74.4%) for proof of vaccination to travel internationally, particularly in China (93.3%), South Korea (87.8%), India (87.7%), Brazil (86.4%), and Ecuador (83%) but less so in Poland (52.7%) and Russia (52.5%). Overall support for mandatory vaccination to attend university and indoor activities was 63.3% and 63.1%, respectively. These ranged from lowest in Russia (33.1% for university attendance and 37.6% for indoor activities) to highest in China (95.4% and 89.7%, respectively). Support for governments or employers to require vaccination displayed similar trends. Agreement with requirements to vaccinate children to attend school received the least support overall (58.2%), ranging from 24.4% in Russia to 86.6% in China. Support for requiring vaccines to participate in situations listed above was substantially lower among those who were hesitant to vaccinate themselves (Fig. 6).

## Discussion

Reported COVID-19 vaccine acceptance, measured by intention to get vaccinated or having received at least one dose of an available vaccine, increased over the last year in 15 of the 19 countries studied in 2020 and 2021^3^, and was 75.2% for all 23 countries studied in 2021. However, this percentage is still below the estimates needed to control the pandemic^29^. Negative perceptions of safety, trust in the science behind vaccine development, and vaccine efficacy were the most consistent correlates of hesitancy. Government mistrust was associated with vaccine hesitancy in most countries. Other factors associated with vaccine hesitancy varied by country and included personal experience with COVID-19 (e.g., sickness or loss of a family member) and demographic characteristics (e.g., gender, education, and income).

In order to improve global vaccination rates, some countries may at present require people to present proof of vaccination to attend work, school, or indoor activities and events. Our results found strong support among participants for requirements targeting international travellers, while support was weakest among participants for requirements for schoolchildren. Support for requiring vaccines was substantially lower among those who were hesitant to get vaccinated themselves. Importantly, however, recommendations by a doctor, or to a lesser extent by an employer, may impact a respondent’s views on vaccination in some countries.

Misperceptions of vaccines as having high risks and low benefits is a driver of vaccine hesitancy^30^ that may also reflect a respondent’s lower trust in the science behind vaccine research and production^21,31–34^. In late 2019 and early 2020 the majority of people surveyed globally reported that science was important in their society. Perceived importance of science was described as valuing government investments in scientific research, believing it is very important to be a world leader in scientific achievement, and trusting scientists to do what is right for the public^35^. Our results corroborate these findings: negative perceptions of trust in science and vaccine safety and efficiacy were most strongly associated with vaccine hesitancy.

Health care workers are in a position of trust and are perceived as important sources of reliable and accurate vaccine information^36–39^. However, one study found that COVID-19 vaccine hesitancy among HCWs ranged widely between 4.3% and 72%, with an average of 22.5%^40^. The main reasons for vaccine hesitancy among HCWs are similar to those found in the general population: concerns about efficacy, safety, and potential side-effects. Demographic factors among HCWs such as male gender, greater age, and attainment of a doctoral degree were positively associated with acceptance of COVID-19 vaccines^40^. Our findings align with prior reports of higher rates of vaccine acceptance among physicians compared to nurses^41–44^. Among hesitant respondents in this survey, the advice of their physician improved willingness to accept a COVID-19 vaccine, as did recommendation by their employer, to a lesser extent.

In June 2020, one of the factors most strongly associated with acceptance of a yet unavailable vaccine was trust in government to successfully address unexpected health threats, such as the COVID-19 pandemic^3^. A 2020 study in Portugal, where 56% of respondents reported they would wait to take the COVID-19 vaccine and 9% of respondents reported they would refuse vaccination, showed that vaccine hesitancy was related to a poor perception of government and health service responses as well as a lack of trust in the information provided^45^, which is consistent with previous pandemic research^46^. Yet, after accounting for socio-demographic and COVID-19 experience variables, our study found mistrust in the central government was not significantly associated with vaccine hesitancy, as was also the case for the local government, in most countries sampled, except in Ghana and Poland. Respondents in Nigeria reported high vaccine hesitancy and high distrust in governmental ability to respond to COVID-19^47^, but the association between vaccine hesitancy and trust in government was not significant. This dissonance between trust in government and vaccine acceptance in our study could be related to the population’s general dissatisfaction with government responses to the pandemic and its economic consequences, with vaccine acceptance being independent of such sentiments and more a reflection of personal experiences with COVID-19 illness or loss of life and livelihood. Our results confirm that direct experience for self or family with the illness and/or loss of a family member to the disease are independently associated with vaccine acceptance.

As health systems in LMICs struggle to address COVID-19^48^, vaccine access has become a cause for national and international frustration. Most LMICs have been slow to receive and distribute vaccines, which are much more available in high-income countries, prompting critiques of global vaccine inequity^49^, which were exacerbated with the distribution of so-called booster shots in high-income countries in the autumn of 2021. Access issues, coupled with vaccine hesitancy, which this study found declined overall, can have catastrophic effects. In South Africa, where hesitancy increased compared to our 2020 study, data collection coincided with the rapid transmission of the Omicron variant. The virulence and transmissibility of future possible variants remain unknown, highlighting the need to augment efforts to increase vaccine equity and public trust in vaccination^50^.

Most recent studies do not investigate income as a potential influencer on vaccine acceptance. A study conducted in Portugal found that people who lost income during the pandemic were more hesitant^45^. A study carried out in Ireland showed that people with a lower income were also more vaccine hesitant^51^. A study carried out in China showed that loss of income was greater among residents of areas with more severe COVID-19 transmission and magnified existing social and economic disparities^52^. A study conducted in the US showed that having lower income was associated with a higher risk of depression during the pandemic^53^. Our results show that a lower household income is associated with a greater level of hesitancy in 11 countries, while a loss of income due to the pandemic is positively associated with vaccine acceptance in four countries. We suspect the perceived positive association between vaccination and desire for a “return to normalcy” is stronger among people who lost socioeconomic status and financial stability due to revenue loss.

Globally, anxiety and depression increased during the pandemic while positive emotions such as happiness and life satisfaction decreased^54^. A global review of COVID-19-related mental health studies found that the prevalence of anxiety ranged between 26.5%–44.6% and depression between 8.1%–25% and that the prevalence of insomnia was 38%^55^. Stress levels were found to vary widely (from 3.8% to 68.3%). In several countries such as Bangladesh, India, and Pakistan, cases of suicide linked to fear of COVID-19 have been reported^54^. Across a range of time points and geographies, people who had COVID-19 reported more symptoms of anxiety, depression, and post-traumatic stress disorder than people without a COVID-19 diagnosis^54^. Similarly, people with pre-existing psychiatric conditions reported a worsening of their psychiatric symptoms during the pandemic^56^.

Mental illness has been associated with a higher risk of COVID-19 related mortality and morbidity^57^, yet studies examining the impact of mental health on vaccine hesitancy are scarce. One German study did not show a relation between depression or anxiety and vaccine hesitancy^58^. This is in line with a Danish study showing that, although people previously diagnosed with mental illnesses reported slightly lower vaccine acceptance compared to the general population (84.8% versus 89.5%), vaccine hesitancy among people with mental illnesses did not seem to be a deterrant to reaching herd immunity^59^. In Ireland, by contrast, people who had received treatment for a mental health problem were more accepting of a vaccine, unlike UK respondents who showed no such association^51^. Our study results suggest that the effects of experience of depression and anxiety are far from universal, with divergent associations between anxiety and depression and vaccine hesitancy reported across the 23 countries. Future research should undertake a deeper examination of cultural influences on mental health-related vaccine hesitancy.

Increased vaccine scepticism may result from the dissemination of erroneous or inaccurate, and often politicized^32,60^, information. Misinformation is associated with vaccine hesitancy, undermining confidence in the safety and efficacy of COVID-19 vaccines^61^. For example, over two-thirds of vaccine related videos on YouTube that were analyzed for content accuracy in May 2019 presented unreliable safety and efficacy information^62^. However, in mid-February 2021, despite only 46% of Twitter poll respondents agreeing that all COVID-19 vaccines are safe, 83% indicated they would accept a vaccine, while only 2% would agree to accept one if mandated^63^. Our results indicate that vaccine hesitant respondents may be more willing to accept a COVID-19 vaccine if recommended by their doctor.

To control the COVID-19 pandemic, some countries have considered or implemented requirements for proof of vaccination, or vaccine mandates, to permit travel internationally or to attend work, school, or indoor events. Consistent with our results, other recent studies found higher support for vaccine requirements to travel internationally than domestically^64,65^. Also in line with our results, a September 2020 study from the US found that approximately half of the general population considered mandatory COVID-19 vaccination for children attending school acceptable, a category that also remains universally low in our sample (overall 58.2%)^66^.

Vaccine mandates for adults by state governments were considered acceptable by 40.9% of the US population, whereas 47.7% accepted mandates by their employer to attend work^66^. However, our 2020 global study showed that people were potentially more likely to accept voluntary over employer-mandated vaccination^3^. “Choice architecture” that frames vaccination requirements as effective public health and disease prevention and control tools, which one chooses to accept in order to fully participate in society, as opposed to a violation of the individual’s right to select medical treatment, may promote incremental vaccine uptake^67^. As vaccines receive full (i.e., no longer just emergency use) approval from regulatory agencies, this may lead to improved perceptions on safety and efficacy, and vaccination campaigns based on such choice framing could convince more unvaccinated adults and young adults to accept vaccination and increase parental acceptance of vaccination for their children.

One limitation of correlation analyses using actual vaccination rates is that countries with low vaccine access may produce unreliable results given this extrinsic factor. Additionally, our questionnaire asked about a general COVID-19 vaccine, whereas several COVID-19 vaccines, each with different efficacy results and targeted misinformation, are being distributed globally. This study is strengthened by maintaining a sampling methodology that ensured population representativeness between iterations. We tested COVID-VAC as a composite score, which showed high internal reliability and external construct validity with our measure for vaccine hesitancy among large nationally representative samples in 23 countries. Yet, other aspects of scale validation have not been conducted, such as test-retest reliability, responsiveness over time, and content validity to assess the potential exclusion of other relevant factors for vaccine hesitancy^68^, though authors consulted the SAGE Working Group framework of determinants and previous related studies to design the questionnaire. Items were written unambiguously and succinctly to adhere to questionnaire development best practices^68^, with the possible exception of item 5, “I trust that my government is able to deliver the COVID-19 vaccine to everyone, everywhere in my country, equally,” which may have introduced a response bias by referring to multiple actors and actions. We encourage further testing of this tool over time, in conjunction with emerging determinants of COVID-19 vaccine hesitancy^69^, and using modified items to reduce potential biases in order to fully understand its validity.

Although some countries are currently disengaging from aggressive COVID-19 control measures, the disease has by no means been subdued. For ongoing COVID-19 vaccination campaigns to succeed in improving coverage going forward, substantial challenges remain to be overcome. These include increasing vaccination among those reporting a lower vaccine confidence or who have difficulties in accessing vaccination services, in addition to expanding vaccine access to low- and middle-income countries. This study confirms the importance of a positive perception of vaccine safety and efficacy for vaccine acceptance under any circumstances. Ongoing vaccination requirements and active vaccine promotion are not without the risk of hardening opposition to COVID-19 vaccination, but the alternative is to risk future surges from new variants and the continuation of the pandemic as a public health threat. Further, misinformation continues to spread and can impact COVID-19 vaccine acceptance^70^. We still need accurate COVID-19 vaccine communication delivered by trusted sources to clearly explain vaccine safety and benefits to individuals, families, and society at large.

## Methods

### Study participants

Participants were recruited by Consensus Strategies using multiple international online panel providers to avoid coverage bias: Dynata provided 22,500 respondents across all 23 countries, and Consensus Strategies provided 500 respondents from Ghana. Participation by unique individuals was ensured by verifying IP addresses and mobile phone numbers. Participants were recruited for the panels via a variety of methods, including email, telephone, and direct mail solicitation and equitably compensated in compliance with ethical standards, varying by country and not exceeding USD 3 per completed survey. Informed consent was obtained from all participants. No personally identifiable information was collected or stored.

### Sampling

Random stratified sampling was employed. Strata included ages (18–29, 30–39, 40–49, 50–59, and 60 years and older); gender (male, female, prefer not to say, and “other”); statistical regions (usually province or state, varies by country); and level of education (based on each country’s educational system^71^), using global data from UNESCO the Organisation for Economic Co-operation and Development, and country data from Sweden, the United Kingdom, and the United States. Educational level was coded into two groups, those who had or had not completed a university degree. The number of participants who could enrol in each of these strata was calculated to reflect the distribution in the general population based on census/survey estimates provided by the World Bank^72^ and the Central Intelligence Agency (CIA) World Factbook^73^. Data were weighted by strata with each stratum requiring a minimum of 50 participants.

### Data collection

Survey data were collected between 25–30 June 2021, from an online panel of 23,000 respondents aged ≥18 years from 23 countries (*n* = 1000 per country), comprised of those countries included in the 2020 study^3^ (*n* = 19), augmented by four additional countries with high disease incidence (Ghana, Kenya, Peru, and Turkey)^74^ and representing regions not represented in the previous study. The 23 countries are: Brazil, Canada, China, Ecuador, France, Germany, Ghana, India, Italy, Kenya, Mexico, Nigeria, Peru, Poland, Russia, Singapore, South Africa, South Korea, Spain, Sweden, Turkey, the UK, and the US. This study was approved and the survey administered by Emerson College, Boston, US (institutional review board protocol no. 20–023-F-E-6/12-[R1] updated April 12, 2021).

### Survey instrument

The instrument was developed by an expert panel following a comprehensive literature review of COVID-19 vaccine acceptance studies and the authors’ earlier studies of pandemic control measures^71,75,76^ and vaccination intent^1,20,30,77–80^, which identified misperceptions of vaccine risk, non-safety and inefficacy, as well as mistrust in government and scientific and health institutions in vaccine development and distribution, as factors associated with vaccine hesitancy. The 31-question instrument (Supplementary Methods) included: 1) questions representing perceptions of risk (q1) (q3), efficacy (q2), safety (q4), and trust (q5 and q6) as important individual determinants of COVID-19 vaccine hesitancy and of routine immunization and explored here as factors in a composite score (COVID-VAC) as well; 2) two vaccine hesitancy-defining questions which included receipt of at least one dose of a COVID-19 vaccine (q7) and hesitancy to take a vaccine when available to them (q8). Vaccine hesitancy was defined as having reported “no” to the question on whether they have received at least one dose of a COVID-19 vaccine and also either “unsure/no opinion,” “somewhat disagree,” or “strongly disagree” to the question on whether they will take COVID-19 vaccine when available to them. COVID-19 vaccine acceptance for self was defined as having received at least one dose of a COVID-19 vaccine and if not, willingness to take the COVID-19 vaccine when it is available (q8 answer options “strongly agree” or “somewhat agree”). In addition, vaccination hesitancy for their children (q9) was defined as having reported “unsure/no opinion,” “somewhat disagree,” or “strongly disagree” to this question on whether they will have their child get the COVID-19 vaccine when it is available to them; 3) vaccination hesitancy if recommended by one’s employer (q10) or doctor (q11); 4) COVID-19 mandate support required by: (a) employers (q12) and (b) the government (q13) and for (c) university students (q14), (d) school children (q15), and (e) indoor activities like auditoriums, concerts, sports events (q16), and (f) international travel (q17); 5) trust in the central (q5 and q24) and local government (q25); 6) experience of anxiety (q21) and depression (q22) (moderate; 3–4 days per week, or most or all of the time; 5–7 days per week); 7) COVID-19 experience (self or a family member became ill with COVID-19 (q19), lost a family member to COVID-19 (q20)); 8) personal or household loss of income due to the COVID-19 pandemic (q18) (Yes, severe loss; Yes, moderate loss; No); and 9) demographic variables (age, gender, education, income) and HCW status.

### Data analysis

This study documents vaccine hesitancy globally and by country at a point in time approximately six months after the first vaccine was authorized and made available for emergency use. Using descriptive statistics, we reported vaccine hesitancy rates by country and globally. The acceptance of a hypothetical vaccine described in our June 2020 study^3^ and current COVID-19 vaccine acceptance now that vaccines are available was also analyzed descriptively. Global estimates were obtained by averaging country-specific estimates. The association between a country’s vaccine hesitancy and COVID-19 cases and mortality (per million population) (Fig. 2)^74^ at the time of the survey were each assessed using Pearson correlations. Weighted univariable and multivariable logistic regressions were used to assess adjusted relationships between vaccine hesitancy and socio-demographic variables, COVID-19 illness experience (personally or a family member), perceptions of trust, safety, and efficacy, trust in the central and local government, and mental health. Associations between vaccine hesitancy and socio-demographic variables were reported as odds ratios (ORs) and associated 95% confidence intervals (CIs).

In addition to adult vaccine hesitancy, we used descriptive statistics to investigate support for vaccination of children and requirements for proof of vaccination to travel internationally or to attend work, school, or indoor events. Finally, vaccine hesitancy among HCWs was assessed across all countries combined. Statistical significance was set at alpha = 0.05. Analyses were conducted in SAS 9.4.

### Reporting summary

Further information on research design is available in the Nature Research Reporting Summary linked to this article.

## Supplementary information

Supplementary Information

Reporting Summary

## Data Availability

The raw data generated in this study are available for download at 10.5281/zenodo.6560427^81^. All authors had access to the raw data. Source data for Fig. 2 and Supplementary Figure 1 are provided with this paper. Data generated by Worldometer (https://www.worldometers.info/coronavirus/about/#sources), the World Bank (https://data.worldbank.org/indicator), and the CIA World Factbook (https://www.cia.gov/the-world-factbook/about/archives) were re-used as described in the Methods. Source data are provided with this paper.

## Source data

Source Data

## Code availability

The analysis code that can be used to replicate the results is available at 10.5281/zenodo.6560427^81^.

## References

[1] MacDonald NE, SAGE Working Group on Vaccine Hesitancy. (2015). Vaccine hesitancy: definition, scope and determinants. Vaccine.

[2] World Health Organization. Ten threats to global health in 2019. https://www.who.int/news-room/spotlight/ten-threats-to-global-health-in-2019 (2019).

[3] Lazarus JV (2020). A global survey of potential acceptance of a COVID-19 vaccine. Nat. Med..

[4] Mathieu, E. et al. A global database of COVID-19 vaccinations. *Nature Human Behaviour 2021 5:7* **5**, 947–953 (2021). 10.1038/s41562-021-01122-8 33972767

[5] Stojanovic J (2021). Global Trends and Correlates of COVID-19 Vaccination Hesitancy: Findings from the iCARE Study. Vaccines 2021.

[6] Boyon, N. COVID-19 vaccination intent is decreasing globally. *Ipsos and World Economic Forum* https://www.ipsos.com/en/global-attitudes-covid-19-vaccine-october-2020 (2020).

[7] Troiano G, Nardi A (2021). Vaccine hesitancy in the era of COVID-19. Public Health.

[8] Sallam M (2021). COVID-19 vaccine hesitancy worldwide: a concise systematic review of vaccine acceptance rates. Vaccines (Basel).

[9] Ratzan SC, Sommariva S, Rauh L (2020). Enhancing global health communication during a crisis: lessons from the COVID-19 pandemic. Public Health Res Pr..

[10] Grossman VA (2021). The COVID-19 vaccine: why the hesitancy?. J. Radiol. Nurs..

[11] Sallam M (2021). Low COVID-19 vaccine acceptance is correlated with conspiracy beliefs among university students in Jordan. Int. J. Environ. Res. Public Health.

[12] Loomba S, de Figueiredo A, Piatek SJ, de Graaf K, Larson HJ (2021). Measuring the impact of COVID-19 vaccine misinformation on vaccination intent in the UK and USA. Nat. Hum. Behav..

[13] Chaccour, C. & Vilasanjuan, R. Infodemic: how has the epidemic of misinformation affected the response to COVID-19? *ISGlobal* (2020).

[14] Puri N, Coomes EA, Haghbayan H, Gunaratne K (2020). Social media and vaccine hesitancy: new updates for the era of COVID-19 and globalized infectious diseases. Hum. Vaccines Immunotherapeutics.

[15] Suzanna Awang, B. et al. Factors affecting COVID-19 vaccine acceptance: an International survey among low- and middle-income countries. *Vaccines (Basel)* **9**, (2021). 10.3390/vaccines9050515 PMC8157062 34067682

[16] Caserotti M (2021). Associations of COVID-19 risk perception with vaccine hesitancy over time for Italian residents. Soc. Sci. Med..

[17] Detoc M (2020). Intention to participate in a COVID-19 vaccine clinical trial and to get vaccinated against COVID-19 in France during the pandemic. Vaccine.

[18] Aw, J., Seng, J., Seah, S. & Low, L. COVID-19 Vaccine hesitancy—a scoping review of literature in high-income Countries. *Vaccines* **9**, 900 (2021). 10.3390/vaccines9080900 PMC8402587 34452026

[19] AlShurman BA, Khan AF, Mac C, Majeed M, Butt ZA (2021). What demographic, social, and contextual factors influence the intention to use COVID-19 vaccines: a scoping review. Int. J. Environ. Res. Public Health.

[20] Karafillakis E, Larson HJ, ADVANCE Consortium. (2017). The benefit of the doubt or doubts over benefits? A systematic literature review of perceived risks of vaccines in European populations. Vaccine.

[21] Palamenghi L, Barello S, Boccia S, Graffigna G (2020). Mistrust in biomedical research and vaccine hesitancy: the forefront challenge in the battle against COVID-19 in Italy. Eur. J. Epidemiol..

[22] de Figueiredo, A. & Larson, H. J. Exploratory study of the global intent to accept COVID-19 vaccinations. *Communications Medicine 2021 1:1* **1**, 1–10 (2021). 10.1038/s43856-021-00027-x PMC9053214 35602227

[23] Malik, A. A., McFadden, S. A. M., Elharake, J. & Omer, S. B. Determinants of COVID-19 vaccine acceptance in the US. *EClinicalMedicine* **26**, (2020). 10.1016/j.eclinm.2020.100495 PMC7423333 32838242

[24] Hamel, L., Kirzinger, A., Muñana, C. & Brodie, M. KFF COVID-19 Vaccine Monitor: December 2020. *Kaiser Family Foundation* https://www.kff.org/coronavirus-covid-19/report/kff-covid-19-vaccine-monitor-december-2020/ (2020).

[25] Callaghan, T. et al. Correlates and Disparities of COVID-19 Vaccine Hesitancy. *SSRN Electronic Journal*10.2139/ssrn.3667971 (2020).

[26] Willis DE (2021). COVID-19 vaccine hesitancy: Race/ethnicity, trust, and fear. Clin. Transl. Sci..

[27] Khubchandani J, Macias Y (2021). COVID-19 vaccination hesitancy in Hispanics and African-Americans: A review and recommendations for practice. Brain Behav. Immun. Health.

[28] World Bank. World Bank Data: Population, total. https://data.worldbank.org/indicator/SP.POP.TOTL.

[29] Kadkhoda K (2021). Herd Immunity to COVID-19. Am. J. Clin. Pathol..

[30] Lane S, MacDonald NE, Marti M, Dumolard L (2018). Vaccine hesitancy around the globe: Analysis of three years of WHO/UNICEF Joint Reporting Form data-2015–2017. Vaccine.

[31] Cadeddu C, Daugbjerg S, Ricciardi W, Rosano A (2020). Beliefs towards vaccination and trust in the scientific community in Italy. Vaccine.

[32] May T (2020). Anti-Vaxxers, Politicization of Science, and the Need for Trust in Pandemic Response. J. Health Commun..

[33] Bicchieri, C. et al. In science we (should) trust: Expectations and compliance across nine countries during the COVID-19 pandemic. *PLoS One* **16**, (2021). 10.1371/journal.pone.0252892 PMC8177647 34086823

[34] Veit W, Brown R, Earp B (2021). In Science We Trust? Being Honest About the Limits of Medical Research During COVID-19. Am. J. Bioeth..

[35] Funk, C., Tyson, A., Kennedy, B. & Johnson, C. *Science and Scientists Held in High Esteem Across Global Publics*. (2020).

[36] Arce, J. S. S. et al. COVID-19 vaccine acceptance and hesitancy in low- and middle-income countries. *Nature Medicine 2021* 1–10 10.1038/s41591-021-01454-y (2021). 10.1038/s41591-021-01454-y PMC8363502 34272499

[37] Yaqub O, Castle-Clarke S, Sevdalis N, Chataway J (2014). Attitudes to vaccination: A critical review. Soc. Sci. Med..

[38] Paterson P (2016). Vaccine hesitancy and healthcare providers. Vaccine.

[39] Verger P (2015). Vaccine Hesitancy Among General Practitioners and Its Determinants During Controversies: A National Cross-sectional Survey in France. EBioMedicine.

[40] Biswas, N., Mustapha, T., Khubchandani, J. & Price, J. H. The Nature and Extent of COVID-19 Vaccination Hesitancy in Healthcare Workers. *Journal of Community Health* 1 10.1007/S10900-021-00984-3 (2021). 10.1007/s10900-021-00984-3 PMC8056370 33877534

[41] Picchio CA (2020). The impact of the COVID-19 pandemic on harm reduction services in Spain. Harm Reduct. J..

[42] Elizondo-Alzola U (2021). Vaccine hesitancy among paediatric nurses: Prevalence and associated factors. PLOS ONE.

[43] Dzieciolowska, S. et al. Covid-19 vaccine acceptance, hesitancy, and refusal among Canadian healthcare workers: A multicenter survey. *American Journal of Infection Control*10.1016/J.AJIC.2021.04.079 (2021). 10.1016/j.ajic.2021.04.079 PMC8079260 33930516

[44] Bauernfeind S (2021). Brief report: attitudes towards Covid-19 vaccination among hospital employees in a tertiary care university hospital in Germany in December 2020. Infection 2021.

[45] Soares P (2021). Factors Associated with COVID-19 Vaccine Hesitancy. Vaccines (Basel).

[46] Siegrist M, Zingg A (2014). The role of public trust during pandemics: Implications for crisis communication. Eur. Psychologist.

[47] Ezeibe, C. C. et al. Political distrust and the spread of COVID-19 in Nigeria. 10.1080/17441692.2020.1828987**15**, 1753–1766 (2020). 10.1080/17441692.2020.1828987 33019916

[48] Okereke M (2021). Impact of COVID-19 on access to healthcare in low- and middle-income countries: Current evidence and future recommendations. Int. J. Health Plan. Manag..

[49] Acharya KP, Ghimire TR, Subramanya SH (2021). Access to and equitable distribution of COVID-19 vaccine in low-income countries. npj Vaccines.

[50] Otto SP (2021). The origins and potential future of SARS-CoV-2 variants of concern in the evolving COVID-19 pandemic. Curr. Biol..

[51] Murphy J (2021). Psychological characteristics associated with COVID-19 vaccine hesitancy and resistance in Ireland and the United Kingdom. Nat. Commun..

[52] Qian Y, Fan W (2020). Who loses income during the COVID-19 outbreak? Evidence from China. Res. Soc. Stratification Mobil..

[53] Ettman, C. et al. Prevalence of Depression Symptoms in US Adults Before and During the COVID-19 Pandemic. *JAMA Netw Open* **3**, (2020). 10.1001/jamanetworkopen.2020.19686 PMC7489837 32876685

[54] Hossain, M. et al. Epidemiology of mental health problems in COVID-19: a review. *F1000Res* **9**, (2020). 10.12688/f1000research.24457.1 PMC7549174 33093946

[55] García-Iglesias J (2020). Impacto del SARS-CoV-2 (Covid-19) en la salud mental de los profesionales sanitarios: una revisión sistemática. Rev. Española de. Salud Pública.

[56] Vindegaard N, Benros M (2020). COVID-19 pandemic and mental health consequences: Systematic review of the current evidence. Brain Behav. Immun..

[57] Mazereel V, Van Assche K, Detraux J, De. Hert M (2021). COVID-19 vaccination for people with severe mental illness: why, what, and how?. Lancet Psychiatry.

[58] Bendau A, Plag J, Petzold MB, Ströhle A (2021). COVID-19 vaccine hesitancy and related fears and anxiety. Int. Immunopharmacol..

[59] Jefsen, O. H. et al. COVID-19 vaccine willingness amongst patients with mental illness compared with the general population. *Acta Neuropsychiatrica* 1–4 10.1017/NEU.2021.15 (2021). 10.1017/neu.2021.15 33998428

[60] Lin C, Tu P, Beitsch LM (2021). Confidence and receptivity for covid‐19 vaccines: A rapid systematic review. Vaccines (Basel).

[61] Islam, M. et al. COVID-19 vaccine rumors and conspiracy theories: The need for cognitive inoculation against misinformation to improve vaccine adherence. *PLoS One* **16**, (2021). 10.1371/journal.pone.0251605 PMC8115834 33979412

[62] Murphy M (2020). Assessing the Validity and Accuracy of Online Videos on Vaccine Health Risks. Clin. Pediatrics.

[63] Eibensteiner, F. et al. People’s Willingness to Vaccinate Against COVID-19 Despite Their Safety Concerns: Twitter Poll Analysis. *Journal of Medical Internet Research* **23**, (2021). 10.2196/28973 PMC8086789 33872185

[64] Smith, D. T., Attwell, K. & Evers, U. Support for a COVID-19 vaccine mandate in the face of safety concerns and political affiliations: An Australian study: *Politics*10.1177/02633957211009066 (2021).

[65] de Figueiredo, A., Larson, H. J. & Reicher, S. D. The potential impact of vaccine passports on inclination to accept COVID-19 vaccinations in the United Kingdom: Evidence from a large cross-sectional survey and modeling study. *EClinicalMedicine* **40**, (2021). 10.1016/j.eclinm.2021.101109 PMC8428473 34522870

[66] Largent, E. A. et al. US Public Attitudes Toward COVID-19 Vaccine Mandates. *JAMA Network Open* **3**, (2020). 10.1001/jamanetworkopen.2020.33324 PMC7749443 33337490

[67] Dubov A, Phung C (2015). Nudges or mandates? The ethics of mandatory flu vaccination. Vaccine.

[68] Boateng GO, Neilands TB, Frongillo EA, Melgar-Quiñonez HR, Young SL (2018). Best Practices for Developing and Validating Scales for Health, Social, and Behavioral Research: A Primer. Front. Public Health.

[69] Recio-Román A, Recio-Menéndez M, Román-González MV (2021). Vaccine Hesitancy and Political Populism. An Invariant Cross-European Perspective. Int. J. Environ. Res. Public Health.

[70] Shakeel, C. S., Mujeeb, A. A., Mirza, M. S., Chaudhry, B. & Khan, S. J. Global COVID-19 Vaccine Acceptance: A Systematic Review of Associated Social and Behavioral Factors. *Vaccines (Basel)* **10**, (2022). 10.3390/vaccines10010110 PMC8779795 35062771

[71] Lazarus JV (2020). COVID-SCORE: A global survey to assess public perceptions of government responses to COVID-19 (COVID-SCORE-10). PLOS ONE.

[72] The World Bank. World Bank Data. https://data.worldbank.org/indicator (2021).

[73] U.S. Central Intelligence Agency. The World Factbook. https://www.cia.gov/the-world-factbook/about/archives/ (2021).

[74] Worldometer. COVID-19 data. https://www.worldometers.info/coronavirus/about/#sources (2020).

[75] Lazarus, J. V et al. Keeping governments accountable: the COVID-19 Assessment Scorecard (COVID-SCORE). *Nature Medicine* 1–4 10.1038/s41591-020-0950-0 (2020). 10.1038/s41591-020-0950-0 32528155

[76] White, T. M. et al. COVID-SCORE Spain: Public perceptions of key government COVID-19 control measures. *European Journal of Public Health*10.1093/eurpub/ckab066 (2021). 10.1093/eurpub/ckab066 PMC8083190 33872348

[77] Larson HJ, Cooper LZ, Eskola J, Katz SL, Ratzan S (2011). Addressing the vaccine confidence gap. Lancet.

[78] Larson HJ (2018). Measuring trust in vaccination: A systematic review. Hum. Vaccines Immunotherapeutics.

[79] Quinn SC (2013). Exploring communication, trust in government, and vaccination intention later in the 2009 H1N1 pandemic: Results of a national survey. Biosecurity Bioterrorism.

[80] Strategic Advisory Group of Experts on Immunization (SAGE). *Report of the SAGE Working Group on Vaccine Hesitancy*. (2014).

[81] White, T.M. Revisiting COVID-19 vaccine hesitancy in 23 countries from 2021 data set. *Zenodo*10.5281/zenodo.6560427 (2022).

