# Supplementary material for: Revisiting COVID-19 vaccine hesitancy around the world using data from 23 countries in 2021
Source: Nat Commun. 2022 Jul 1;13:3801. doi: 10.1038/s41467-022-31441-x (PMC9247969; doi:10.1038/s41467-022-31441-x)
Supplement: Supplementary file 1 — Supplementary Information [file 41467_2022_31441_MOESM1_ESM.pdf]

Supplementary Information

Supplementary Methods

1. Survey Instrument

Title: COVID-VAC: A global survey of COVID-19 vaccine perceptions

The purpose of the survey is to measure resident's reactions to COVID-19 response efforts. The risks in this routine survey are considered minimal. You may feel uncomfortable considering the implications of COVID-19 and participation in this research study is completely voluntary and you may discontinue participation at any time. Your responses will remain anonymous and confidential. The survey should take less than 5 minutes to complete. By answering the questions you are indicating that you have read the description of the study, are over the age of 18, and that you agree to the terms as described.

If you have questions about your rights as a research participant, you may contact the Emerson College Institutional Review Board (IRB), which is concerned with the protection of volunteers in research projects. You may reach the lead investigator via email at emersonpolling@emerson.edu or the Chair of the IRB by e-mailing human_subjects@emerson.edu.

1. COVID-19 is a dangerous health threat.
    a. Strongly agree
    b. Somewhat agree
    c. Unsure/no opinion
    d. Somewhat disagree
    e. Strongly disagree

2. COVID-19 can be prevented by vaccination.
    a. Strongly agree
    b. Somewhat agree
    c. Unsure/no opinion
    d. Somewhat disagree
    e. Strongly disagree

3. The risks of COVID-19 disease are greater than the risks of the vaccine
    a. Strongly agree
    b. Somewhat agree
    c. Unsure/no opinion
    d. Somewhat disagree
    e. Strongly disagree

4. The COVID-19 vaccines available to me are safe

    a. Strongly agree
    b. Somewhat agree
    c. Unsure/no opinion
    d. Somewhat disagree
    e. Strongly disagree

5. I trust that my government is able to deliver the COVID-19 vaccine to everyone, everywhere in my country, equally.
    a. Strongly agree
    b. Somewhat agree
    c. Unsure/no opinion
    d. Somewhat disagree
    e. Strongly disagree

6. I trust the science behind the COVID-19 vaccines.
    a. Strongly agree
    b. Somewhat agree
    c. Unsure/no opinions
    d. Somewhat disagree
    e. Strongly disagree

7. Have you received at least one dose of a COVID-19 vaccine?
    a. Yes (Skip to question 9)
    b. No

8. I will take the COVID-19 vaccine when it is available to me.
    a. Strongly agree
    b. Somewhat agree
    c. Unsure/no opinions
    d. Somewhat disagree
    e. Strongly disagree

9. I will have my child get the COVID-19 vaccine when it is available for them.
    a. Strongly agree
    b. Somewhat agree
    c. Unsure/no opinions
    d. Somewhat disagree
    e. Strongly disagree
    f. I do not have a child

10. I will take the COVID-19 vaccine if my employer recommends it.
    a. Strongly agree

    b. Somewhat agree
    c. Unsure/no opinions
    d. Somewhat disagree
    e. Strongly disagree

11. I will take the COVID-19 vaccine if my doctor recommends it.
    a. Strongly agree
    b. Somewhat agree
    c. Unsure/no opinions
    d. Somewhat disagree
    e. Strongly disagree

12. Employers should require that their employees take a COVID-19 vaccine.
    a. Strongly agree
    b. Somewhat agree
    c. Unsure/no opinions
    d. Somewhat disagree
    e. Strongly disagree

13. The government should require people to take a COVID-19 vaccine.
    a. Strongly agree
    b. Somewhat agree
    c. Unsure/no opinions
    d. Somewhat disagree
    e. Strongly disagree

14. Universities should require students to take a COVID-19 vaccine.
    a. Strongly agree
    b. Somewhat agree
    c. Unsure/no opinions
    d. Somewhat disagree
    e. Strongly disagree

15. School children should be required to take a COVID-19 vaccine.
    a. Strongly agree
    b. Somewhat agree
    c. Unsure/no opinions
    d. Somewhat disagree
    e. Strongly disagree

16. Proof of vaccination should be required to enter indoor activities like movie theaters, concerts, and sports arenas.

a. Strongly agree
b. Somewhat agree
c. Unsure/no opinions
d. Somewhat disagree
e. Strongly disagree

17. Proof of vaccination should be required for international travel.
    a. Strongly agree
    b. Somewhat agree
    c. Unsure/no opinions
    d. Somewhat disagree
    e. Strongly disagree

18. Have you or anyone else in your household experienced a loss in income due to the COVID-19 pandemic?
    a. Yes, a severe loss
    b. Yes, a moderate loss
    c. No

19. Did you or someone in your family get sick with COVID-19?
    a. Yes
    b. No (***skip to Q21***)

20. Have you lost a family member to COVID-19 disease?
    a. Yes
    b. No

21. In the past week, how often have you felt nervous, anxious, or on edge?
    a. Rarely or none of the time (less than 1 day);
    b. Some or a little of the time (1-2 days);
    c. Occasionally or a moderate amount of time (3-4 days);
    d. Most or all of the time (5-7 days)

22. In the past week, have you felt down, depressed, or hopeless?
    a. Rarely or none of the time (less than 1 day);
    b. Some or a little of the time (1-2 days);
    c. Occasionally or a moderate amount of time (3-4 days);
    d. Most or all of the time (5-7 days)

23. Are you one of the following?
    a. Physician
    b. Nurse

c. Community Health Worker
d. Other healthcare worker
e. None of the above

24. Do you trust that your central government will successfully address unexpected health threats to our nation, including COVID-19 pandemic?
    a. Yes
    b. No

25. Do you trust that your local government will successfully address unexpected health threats to our nation, including COVID-19 pandemic?
    a. Yes
    b. No

26. Generally speaking, would you say that most people can be trusted or that you need to be very careful in dealing with people?
    1. Yes, most people can be trusted
    2. No, you need to be very careful in dealing with people

27. What is your age?

28. What is your gender?
    a. Male
    b. Female
    c. Prefer not to say
    d. Other

29. Education (country-specific)

30. Income (country-specific)

31. Regions (country-specific)

Supplementary Table 1. COVID-19 vaccine acceptance by individual COVID-VAC questions.

	COVID-19 is a dangerous health threat.		COVID-19 can be prevented by vaccination.		The risks of COVID-19 disease are greater than the risks of the vaccine		The COVID-19 vaccines available to me are safe		I trust that my government is able to deliver the COVID-19 vaccine to everyone, everywhere in my country, equally.		I trust the science behind the COVID-19 vaccines.	
	OR	95%CI	OR	95%CI	OR	95%CI	OR	95%CI	OR	95%CI	OR	95%CI
Brazil	3.1	(1.36-7.06)	3.8	(2.6-5.56)	2.19	(1.65-2.92)	4.07	(2.29-7.24)	1.16	(0.82-1.64)	6.64	(3.56-12.41)
Canada	2.76	(2.22-3.42)	3.92	(3-5.14)	4.51	(3.32-6.12)	10.3	(5.72-18.55)	2.81	(2.29-3.44)	5.89	(4.25-8.16)
China	0.62	(0.13-3.02)	1.11	(0.3-4.08)	2.41	(0.99-5.84)	6.17	(0.99-38.37)	2.33	(0.58-9.39)	17.1	(3.27-89.91)
Ecuador	1.98	(0.57-6.94)	4.57	(2.68-7.8)	4.44	(2.36-8.35)	7.39	(3.18-17.18)	1.22	(0.59-2.55)	5.91	(2.85-12.25)
France	2.21	(1.87-2.61)	5.38	(4.11-7.03)	4.33	(3.44-5.46)	9.12	(6.46-12.89)	2.8	(2.38-3.28)	7.15	(5.44-9.39)
Germany	2.47	(2.03-3)	3.52	(2.84-4.35)	3.7	(2.95-4.65)	4.47	(3.45-5.79)	2.26	(1.93-2.64)	4.83	(3.82-6.1)
Ghana	4.55	(1.21-17.1)	3.85	(1.54-9.59)	2.49	(1.34-4.65)	4.65	(1.68-12.86)	1.51	(0.94-2.41)	3.93	(2.21-7.01)
India	1.85	(0.69-4.98)	19.59	(5.61-68.41)	1.8	(0.62-5.17)	3.62	(1.1-11.9)	3.34	(1.9-5.84)	13.1	(1.14-149.4)
Italy	2.87	(2.29-3.6)	6.88	(4.96-9.53)	4.11	(3.2-5.27)	7.97	(5.4-11.77)	3.04	(2.39-3.87)	5.93	(4.33-8.13)
Kenya	1.43	(0.78-2.65)	2.06	(1.35-3.15)	1.66	(1.15-2.4)	3.89	(2.36-6.41)	1.68	(1.28-2.21)	2.35	(1.53-3.63)
Mexico	1.93	(1.22-3.06)	3.71	(2.44-5.64)	1.88	(1.3-2.73)	6.14	(3.47-10.86)	1.67	(1.27-2.19)	4.96	(3.05-8.05)
Nigeria	2.25	(0.8-6.33)	6.88	(3.56-13.27)	2.62	(1.59-4.33)	3.47	(1.48-8.1)	1.77	(1.08-2.88)	4.37	(2.6-7.36)
Peru	2.46	(1.64-3.68)	2.77	(2.08-3.68)	2.25	(1.73-2.92)	10.16	(6.18-16.7)	1.52	(1.09-2.12)	5.71	(3.55-9.18)
Poland	2.53	(2.05-3.13)	4.39	(3.52-5.47)	3.94	(3.22-4.81)	4.79	(3.89-5.9)	2.36	(1.98-2.8)	3.97	(3.26-4.83)
Russia	2.04	(1.49-2.8)	4.07	(3.11-5.33)	3.91	(3.06-5)	4.65	(3.55-6.09)	1.9	(1.54-2.34)	3.54	(2.73-4.59)
South Africa	2.13	(1.4-3.26)	2.37	(1.94-2.9)	2.74	(2.22-3.39)	5.55	(4.07-7.58)	1.97	(1.68-2.3)	4.19	(3.28-5.35)
South Korea	1.54	(1.29-1.82)	2.41	(2.01-2.9)	2.06	(1.75-2.43)	3.53	(2.86-4.36)	2.14	(1.85-2.49)	3.36	(2.71-4.18)
Singapore	1.54	(1.14-2.07)	2.69	(2.06-3.51)	2.45	(1.9-3.17)	4.79	(3.26-7.04)	2.87	(2.01-4.12)	4.95	(3.42-7.16)
Spain	3.16	(2.37-4.21)	4.89	(3.65-6.56)	4.64	(3.43-6.29)	8	(5.67-11.29)	2.76	(2.27-3.35)	5.71	(4.24-7.68)
Sweden	2.69	(2.02-3.58)	4	(2.97-5.37)	2.95	(2.3-3.79)	4.69	(3.42-6.45)	2.44	(2-2.98)	3.59	(2.91-4.43)
Turkey	2.18	(1.52-3.11)	3.29	(2.02-5.35)	3.27	(2.28-4.68)	7.55	(4.51-12.64)	2.37	(1.68-3.34)	4.97	(3.2-7.71)
United Kingdom	2.3	(1.85-2.86)	3.44	(2.76-4.29)	3.58	(2.79-4.59)	6.74	(4.68-9.7)	2.64	(2.09-3.33)	5.05	(3.58-7.13)
United States	2.56	(2.14-3.06)	4.62	(3.44-6.2)	2.77	(2.19-3.52)	4.39	(3.09-6.22)	2.75	(2.22-3.39)	3.89	(3-5.05)
**All countries combined**	**2.16**	**(2.08-2.23)**	**3.61**	**(3.47-3.74)**	**2.97**	**(2.87-3.06)**	**5.3**	**(5.06-5.55)**	**2.03**	**(1.98-2.08)**	**4.51**	**(4.33-4.70)**
**Cronbach's alpha (all items)**	**0.86**											

Legend: Unadjusted odds ratios (OR) and 95%CI are from weighted univariable logistic regression.

Supplementary Figure 1. COVID-19 vaccine acceptance and COVID-VAC association

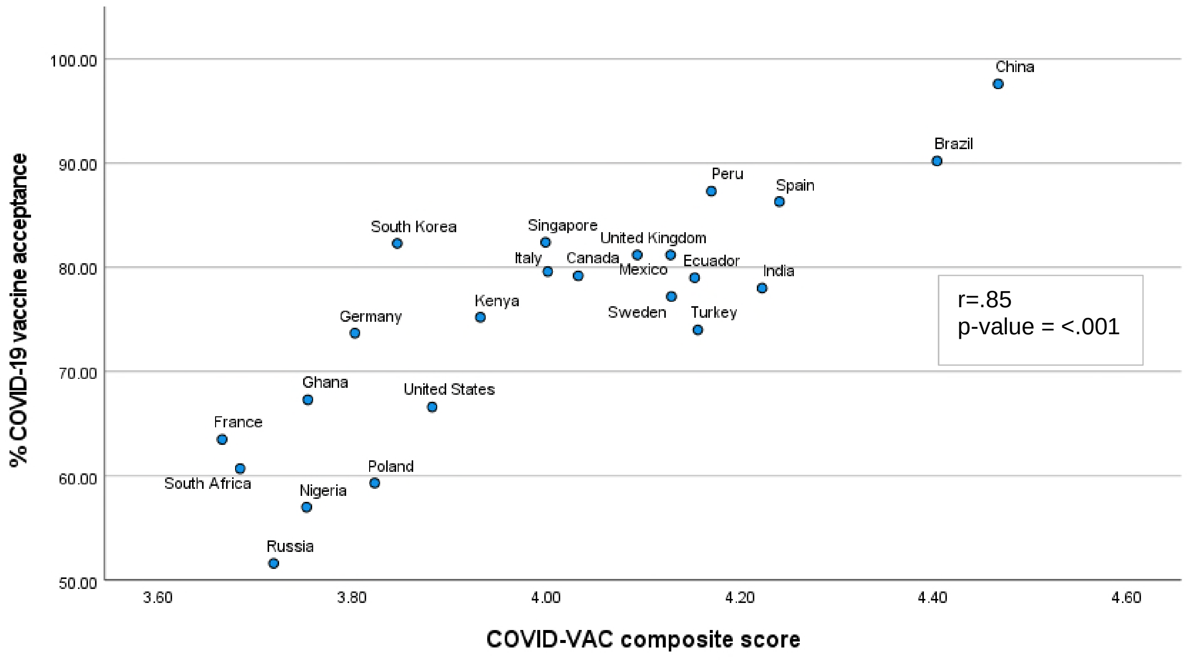

Legend. COVID-19 vaccine acceptance in June 2021 was defined as having received at least one dose of a COVID-19 vaccine and if not, willingness to take the COVID-19 vaccine when it is available to them. COVID-VAC composite score is averaged scores for the six items assessing vaccine-specific perceptions of risk, trust, safety, and efficacy. The association between a country's COVID-19 vaccine acceptance and COVID-VAC composite score were assessed using Pearson correlation and associated p-value based on a two-sided test. Source data are provided in a Source Data file.

Supplementary Figure 2. Trust in central and local government in June 2020 and June 2021.

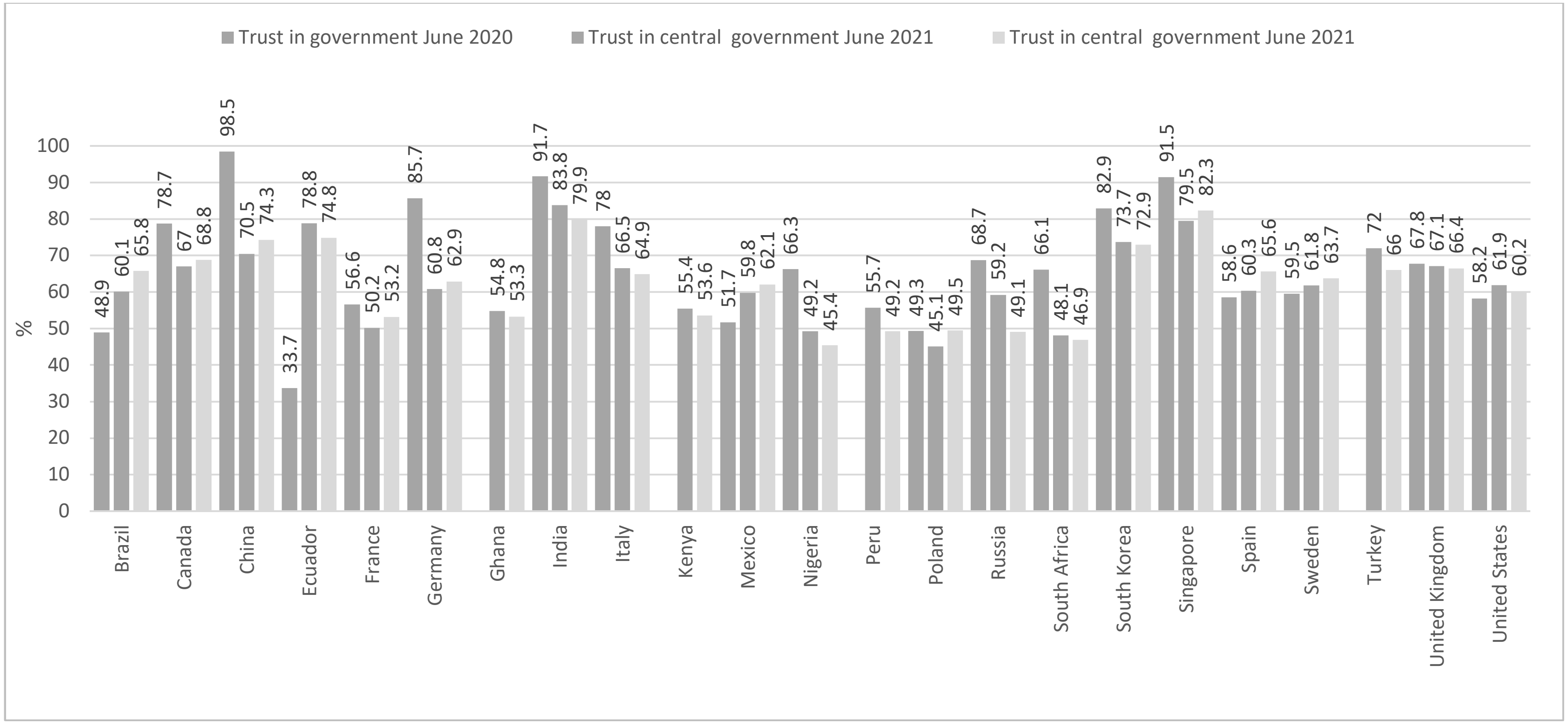

Legend: Trust in government in June 2020 was assessed using the following question: Do you trust that your government will successfully address unexpected health threats to our nation, including COVID 19 epidemic? (Yes/No); Trust in central /local government in June 2020 was assess using the following question(s): Do you trust that your central/local government will successfully address unexpected health threats to our nation, including COVID-19 pandemic? (Yes/No).

Supplementary Table 2. COVID-19 vaccine acceptance by trust in central and local government.

	Trust in central government						Trust in local government					
	Trust in central government	Vaccine acceptance					Trust in local government	Vaccine acceptance				
	%Yes	%Yes	%No	OR	95%CI		%Yes	%Yes	%No	OR	95%CI	
Brazil	60.1	88.7	92.5	0.63	0.27	1.48	65.8	91.5	87.7	1.52	0.65	3.58
**Canada**	**67**	**91.6**	**53.9**	**9.32**	**5.57**	**15.61**	**68.8**	**90.7**	**53.8**	**8.31**	**5.04**	**13.73**
China	70.5	97.0	98.6	0.49	0.08	3.02	74.3	97.2	98.4	0.58	0.09	3.67
Ecuador	78.8	82.9	64.2	2.82	0.68	11.66	74.8	81.7	71.0	1.9	0.48	7.59
**France**	**50.2**	**83.5**	**43.4**	**6.53**	**4.61**	**9.25**	**53.2**	**82.1**	**42.2**	**6.29**	**4.48**	**8.83**
**Germany**	**60.8**	**87.2**	**52.8**	**6.01**	**3.96**	**9.12**	**62.9**	**87.7**	**50.0**	**7**	**4.6**	**10.67**
**Ghana**	**54.8**	**90.7**	**38.9**	**16.01**	**4.66**	**54.99**	**53.3**	**89.3**	**42.2**	**11.74**	**3.51**	**39.21**
**India**	**83.8**	**87.0**	**31.1**	**14.95**	**2.3**	**97.28**	**79.9**	**91.6**	**23.9**	**36.01**	**4.25**	**304.8**
**Italy**	**66.5**	**91.0**	**56.7**	**7.82**	**5.07**	**12.05**	**64.9**	**89.5**	**61.1**	**5.51**	**3.64**	**8.35**
**Kenya**	**55.4**	**86.8**	**60.8**	**4.2**	**1.83**	**9.61**	**53.6**	**88.6**	**59.7**	**5.04**	**2.33**	**10.89**
**Mexico**	**59.8**	**91.1**	**66.4**	**5.18**	**2.28**	**11.79**	**62.1**	**92.3**	**62.9**	**7.05**	**3.11**	**16**
**Nigeria**	**49.2**	**79.1**	**35.6**	**6.75**	**1.62**	**28.04**	**45.4**	**81.5**	**36.7**	**7.5**	**1.69**	**33.24**
Peru	55.7	89.9	84.0	1.61	0.71	3.62	49.2	89.4	85.3	1.37	0.6	3.14
**Poland**	**45.1**	**74.7**	**46.7**	**3.32**	**2.42**	**4.57**	**49.5**	**78.0**	**41.0**	**5.07**	**3.65**	**7.03**
**Russia**	**59.2**	**67.2**	**28.9**	**4.99**	**3.32**	**7.49**	**49.1**	**64.8**	**38.9**	**2.87**	**1.96**	**4.22**
**South Africa**	**48.1**	**76.9**	**45.7**	**3.92**	**2.62**	**5.87**	**46.9**	**74.2**	**48.8**	**2.96**	**1.99**	**4.41**
**South Korea**	**73.7**	**88.1**	**65.8**	**3.74**	**2.61**	**5.36**	**72.9**	**87.9**	**66.9**	**3.52**	**2.46**	**5.04**
**Singapore**	**79.5**	**90.3**	**51.7**	**8.54**	**4.75**	**15.37**	**82.3**	**88.3**	**54.8**	**6.23**	**3.48**	**11.18**
**Spain**	**60.3**	**96.5**	**70.8**	**11.22**	**5.63**	**22.35**	**65.6**	**95.6**	**68.6**	**10.07**	**5.6**	**18.11**
**Sweden**	**61.8**	**87.2**	**61.0**	**4.38**	**2.83**	**6.77**	**63.7**	**86.2**	**61.2**	**4.16**	**2.7**	**6.42**
**Turkey**	**72**	**80.9**	**56.4**	**3.39**	**1.55**	**7.44**	**66**	**83.3**	**55.9**	**4.19**	**1.97**	**8.88**
**United Kingdom**	**67.1**	**90.9**	**61.4**	**6.43**	**3.97**	**10.42**	**66.4**	**92.0**	**59.8**	**8.03**	**4.87**	**13.24**
**United States**	**61.9**	**84.2**	**38.1**	**8.71**	**5.75**	**13.19**	**60.2**	**83.4**	**41.3**	**7.13**	**4.73**	**10.74**

Legend: Unadjusted odds ratios (OR) and 95%CI are from weighted univariable logistic regression; reference category: no trust in government.

Supplementary Figure 3. Reported experience of loss of income.

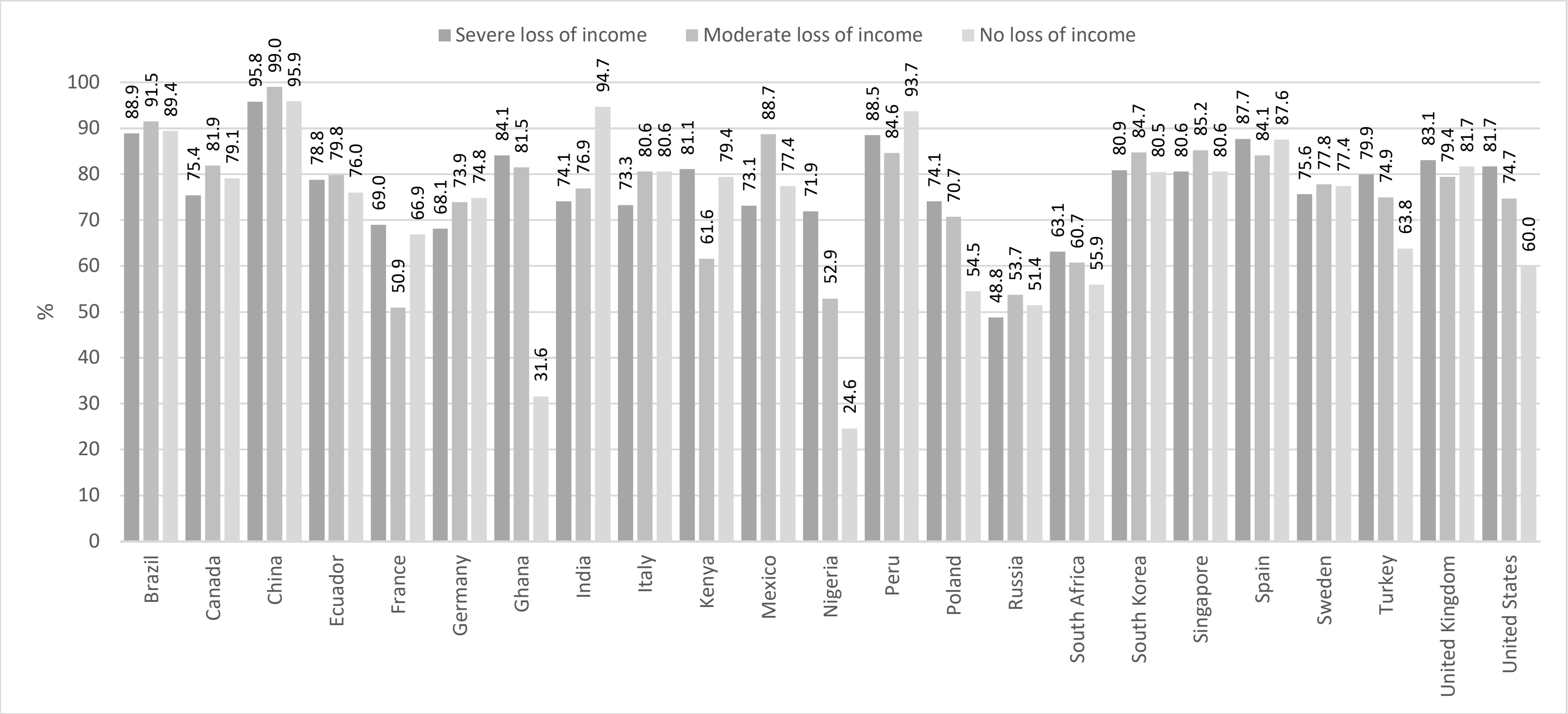

Legend: Loss of income was assessed using the following question: Have you or anyone else in your household experienced a loss in income due to the COVID-19 pandemic? (Yes, a severe loss/Yes, a moderate loss/No)

Supplementary Table 3. COVID-19 vaccine acceptance by experience of anxiety and depression.

	In the past week, how often have you felt nervous, anxious, or on edge?						In the past week, have you felt down, depressed, or hopeless?					
	Overall prevalence	Vaccine acceptance					Overall Prevalence	Vaccine acceptance				
	% 3+days	%3+ days	%<3 days	OR	95%CI		% 3+days	%3+ days	%<3 days	OR	95%CI	
Brazil	25.1	92.0	89.6	1.32	0.5	3.53	25.3	88.1	90.8	0.76	0.31	1.87
Canada	24.9	81.9	78.3	1.26	0.77	2.08	23.1	75.4	80.3	0.75	0.46	1.24
China	12.3	100.0	97.3	-	-	-	12.6	100	97.1	-	-	-
Ecuador	21.7	86.6	76.9	1.88	0.47	7.53	23.1	83.5	77.6	1.73	0.43	7
**France**	**28.7**	**55.4**	**66.7**	**0.63**	**0.45**	**0.87**	**23.1**	**55.7**	**65.8**	**0.65**	**0.46**	**0.92**
**Germany**	**20.6**	**63.6**	**76.3**	**0.56**	**0.36**	**0.86**	**19.2**	**61.5**	**76.6**	**0.51**	**0.33**	**0.79**
Ghana	9.2	61.5	67.8	0.75	0.24	2.36	12.9	69.0	67.0	1.07	0.28	3.99
India	26.7	67.8	81.8	0.45	0.06	3.31	15	93.3	75.3	4.36	0.99	19.19
Italy	26.1	81.2	79.0	1.12	0.7	1.78	22.4	78.1	79.9	0.88	0.55	1.41
Kenya	24.4	73.1	75.9	0.84	0.31	2.3	32.5	72.3	76.6	0.78	0.33	1.86
Mexico	29.4	85.0	79.6	1.45	0.53	4.01	23.8	84.5	80.2	1.32	0.43	3.99
Nigeria	13.3	78.9	53.6	3.22	0.67	15.59	25.1	70.5	52.5	2.16	0.41	11.29
**Peru**	**39.3**	**92.1**	**84.2**	**2.4**	**1.07**	**5.36**	28.8	87.2	87.4	0.97	0.43	2.21
Poland	20.6	54.4	60.6	0.76	0.54	1.08	**20.3**	**51.7**	**61.2**	**0.67**	**0.47**	**0.95**
Russia	36.6	47.8	53.8	0.8	0.55	1.15	27.2	47.6	53.1	0.8	0.53	1.2
South Africa	37	62.7	59.5	1.15	0.78	1.69	30.9	56.6	62.4	0.77	0.51	1.15
**South Korea**	**22.7**	**72.6**	**85.1**	**0.47**	**0.32**	**0.67**	21.1	77.3	83.6	0.68	0.46	1.01
Singapore	20.8	86.1	81.4	1.43	0.69	2.95	21.6	86.0	81.4	1.41	0.69	2.87
Spain	24.4	85.2	86.7	0.9	0.51	1.58	22.2	83.8	87.0	0.81	0.45	1.45
Sweden	24.1	72.6	78.6	0.74	0.47	1.16	**23.8**	**69.7**	**79.5**	**0.6**	**0.38**	**0.94**
Turkey	44.7	70.2	77.2	0.66	0.31	1.4	38.7	69.1	77.2	0.62	0.29	1.33
United Kingdom	27.6	76.7	83.0	0.67	0.42	1.06	25.9	79.2	81.9	0.83	0.52	1.33
**United States**	**21.9**	**56.6**	**69.4**	**0.58**	**0.37**	**0.89**	23.2	60.2	68.6	0.69	0.45	1.08

Legend: Experience of anxiety and depression was defined as symptoms moderate amount of time (3-4 days) or most or all of the time (5-7 days). Unadjusted odds ratios (OR) and 95%CI are from weighted univariable logistic regression; reference category: < 3 days.

Supplementary Table 4. Health care worker (HCW) COVID-19 vaccine acceptance by demographics and COVID-19 experience.

	HCW					Non-HCW				
	Total	At least one dose received	Will take vaccine when available	Vaccine hesitancy	Vaccine acceptance	Total	At least one dose received	Will take vaccine when available	Vaccine hesitancy	Vaccine acceptance
	%	%	%	%	%	%	%	%	%	
Overall	--	72.4	19.4	8.1	91.8	--	52	31	17	83
Age groups										
18-29	37.7	59.3	30.9	9.7	90.2	28.2	29.3	50.7	20	80
30-39	31.1	76.4	16.4	7.2	92.8	22.5	41.6	37.6	20.8	79.2
40-49	12.3	79.5	12.3	8.1	91.8	14.2	53.8	27.8	18.3	81.6
50-59	9.7	85.2	6.3	8.5	91.5	14.5	63.9	21.8	14.3	85.7
60+	9.2	89.8	5.9	4.3	95.7	20.7	83.4	6.3	10.3	89.7
Sex										
Male	49	74	19	7	93	50.3	54.1	30.5	15.4	84.6
Female	49.9	70.8	20	9.2	90.8	49.3	49.5	31.9	18.6	81.4
Income (country median)										
More than median	61.2	81.4	13.3	5.3	94.7	43.8	58.7	28.5	12.9	87.2
Less than median	31.8	61.8	26.7	11.5	88.5	43.5	51.6	29.4	19	81
No income	7	42.7	39.2	18.1	81.9	12.8	28.8	46.3	24.9	75.1
Education (University degree)										
No	39.3	67.4	21.5	11.1	88.9	51.3	50.1	30	19.9	80.1
Yes	60.7	75.7	18.1	6.2	93.8	48.7	53.5	32.4	14.1	85.9
COVID-19 exp										
None	42.8	62.7	24.4	13	87.1	68.8	54.1	28	17.8	82.1
Self/family member sick	19.2	66.1	24.9	9	91	22	46.9	35.3	17.8	82.2
Lost family member	38	86.7	11.1	2.2	97.8	9.2	45.7	44.4	9.9	90.1

Legend: HCW were significantly older (particularly in 50+ age groups, p<.001), similar in terms of sex (p>.05), and had higher income and education. They also reported more severe COVID-19 experience (lost family member, p<.001). After adjusting for demographic variables, COVID-19 experience and clustering of HWC in countries, HCW were more likely to accept vaccine (at least one dose or willing to take when available) (aOR=1.72, 95%CI (1.38, 2.13).
