# Supplementary material for: Revisiting COVID-19 vaccine hesitancy around the world using data from 23 countries in 2021
Source: Nat Commun. 2022 Jul 1;13:3801. doi: 10.1038/s41467-022-31441-x (PMC9247969; doi:10.1038/s41467-022-31441-x)
Supplement: Supplementary file 2 — Reporting Summary [file 41467_2022_31441_MOESM2_ESM.pdf]

nature portfolio

Corresponding author(s): Jeffrey V. Lazarus

Last updated by author(s): Jun 14, 2022

Reporting Summary

Nature Portfolio wishes to improve the reproducibility of the work that we publish. This form provides structure for consistency and transparency in reporting. For further information on Nature Portfolio policies, see our Editorial Policies and the Editorial Policy Checklist.

Statistics

For all statistical analyses, confirm that the following items are present in the figure legend, table legend, main text, or Methods section.

n/a	Confirmed	
☐	☒	The exact sample size (n) for each experimental group/condition, given as a discrete number and unit of measurement
☐	☒	A statement on whether measurements were taken from distinct samples or whether the same sample was measured repeatedly
☐	☒	The statistical test(s) used AND whether they are one- or two-sided<br>*Only common tests should be described solely by name; describe more complex techniques in the Methods section.*
☐	☒	A description of all covariates tested
☐	☒	A description of any assumptions or corrections, such as tests of normality and adjustment for multiple comparisons
☐	☒	A full description of the statistical parameters including central tendency (e.g. means) or other basic estimates (e.g. regression coefficient) AND variation (e.g. standard deviation) or associated estimates of uncertainty (e.g. confidence intervals)
☐	☒	For null hypothesis testing, the test statistic (e.g. F, t, r) with confidence intervals, effect sizes, degrees of freedom and P value noted<br>*Give P values as exact values whenever suitable.*
☒	☐	For Bayesian analysis, information on the choice of priors and Markov chain Monte Carlo settings
☒	☐	For hierarchical and complex designs, identification of the appropriate level for tests and full reporting of outcomes
☐	☒	Estimates of effect sizes (e.g. Cohen's d, Pearson's r), indicating how they were calculated

Our web collection on statistics for biologists contains articles on many of the points above.

Software and code

Policy information about availability of computer code

Data collection	Authors used no software for data collection.
Data analysis	Analyses were conducted in SAS 9.4.

For manuscripts utilizing custom algorithms or software that are central to the research but not yet described in published literature, software must be made available to editors and reviewers. We strongly encourage code deposition in a community repository (e.g. GitHub). See the Nature Portfolio guidelines for submitting code & software for further information.

Data

Policy information about availability of data

All manuscripts must include a data availability statement. This statement should provide the following information, where applicable:

- Accession codes, unique identifiers, or web links for publicly available datasets
- A description of any restrictions on data availability
- For clinical datasets or third party data, please ensure that the statement adheres to our policy

The raw data generated in this study have been deposited and are available for download in the GitHub data repositoryat (https://doi.org/10.5281/zenodo.6560427. All authors had access to the raw data. Source data for Figure 2 and Supplementary Figure 1 are provided with this paper. Data generated by Worldometer (https://www.worldometers.info/coronavirus/about/#sources), the World Bank (https://data.worldbank.org/indicator), and CIA World Factbook (https://www.cia.gov/the-world-factbook/about/archives) were re-used as described in the Methods.

Field-specific reporting

Please select the one below that is the best fit for your research. If you are not sure, read the appropriate sections before making your selection.

- [ ] Life sciences
- [x] Behavioural & social sciences
- [ ] Ecological, evolutionary & environmental sciences

For a reference copy of the document with all sections, see nature.com/documents/nr-reporting-summary-flat.pdf

Behavioural & social sciences study design

All studies must disclose on these points even when the disclosure is negative.

Study description	Cross-sectional random sample general population survey of 23,000 individuals in 23 countries
Research sample	23,000 members of the general public, aged 18 or older, representative of the
Sampling strategy	Strata were established by age (using the following age groups: 18–24, 25–54, 55–64 and 65 years and older); gender (male, female, transgender, and “other,”); and level of education (based on each country’s educational system), which was calculated from data provided by UNESCO, the Organisation for Economic Co-operation and Development, and country data from Sweden, the United Kingdom, and the United States. Educational level was coded into three groups of low, medium and high. “Low” included people who reported not finishing a secondary education (high school); “medium” included those who had completed secondary, vocational, technical, professional associate or high school degree; the “high” group consisted of those who had completed a tertiary or bachelor’s degree and postgraduate work. Each country was divided into regions based on city/town, province or state unit of analysis. The number of participants who could enrol in each of these strata was calculated to reflect the distribution in the general population based on census/survey estimates provided by the World Bank and CIA World Factbook. Data were weighted by strata with each stratum requiring a minimum of 50 participants.
Data collection	Online panels provided responses from 23,000 respondents aged >18 years from 23 countries (n=1,000 per country), comprised of those countries included in the 2020 study (n=19) , augmented by four additional countries with high disease incidence (Ghana, Kenya, Peru, and Turkey) and representing regions not represented in the previous study. The 23 countries are: Brazil, Canada, China, Ecuador, France, Germany, Ghana, India, Italy, Kenya, Mexico, Nigeria, Peru, Poland, Russia, Singapore, South Africa, South Korea, Spain, Sweden, Turkey, the United Kingdom (UK) and the United States (US).
Timing	Survey data were collected between 25-30 June 2021
Data exclusions	Based on sampling strategy, if a stratum was full or if participants did not meet the minimum age requirement
Non-participation	Incomplete responses were not included per this methodology
Randomization	Stratified random sampling was employed. For each demographic stratum, a minimum of 50 responses were established as a quorum. Beyond this minimum quorum, target probabilities were established for each stratum, working backward from 1000 total responses for each country, to equal the country's characteristics, as described in the Sampling strategy above. Respondents were then randomly selected within each stratum. For example, if 51% of a country's demography is female, 510 responses were reserved for females.

Reporting for specific materials, systems and methods

We require information from authors about some types of materials, experimental systems and methods used in many studies. Here, indicate whether each material, system or method listed is relevant to your study. If you are not sure if a list item applies to your research, read the appropriate section before selecting a response.

Materials & experimental systems

n/a	Involved in the study	
x		Antibodies
x		Eukaryotic cell lines
x		Palaeontology and archaeology
x		Animals and other organisms
	x	Human research participants
x		Clinical data
x		Dual use research of concern

Methods

n/a	Involved in the study	
x		ChIP-seq
x		Flow cytometry
x		MRI-based neuroimaging

Human research participants

Policy information about studies involving human research participants

Population characteristics	Demographic information (i.e., age, gender, income, and education) were collected for all participants as well as country of response. Strata for these characteristics were established to ensure that, for each country, the sample population characteristics represent the country's general population.
Recruitment	Participants were recruited through international online panel providers via online email address, telephone and direct mail solicitation. Unique responses were verified using respondent IP addresses or mobile phone numbers to ensure that each participant was real and unique upon registration. Self-selection bias may have been present using this methodology, which could impact the results if people who are are more or less likely to be vaccine hesitant were motivated to respond, or not (non-response bias) to the survey upon learning this focus.
Ethics oversight	This study was approved and the survey administered by Emerson College, Boston, USA (institutional review board protocol no. 20–023-F-E-6/12-[R1] updated April 12, 2021).

Note that full information on the approval of the study protocol must also be provided in the manuscript.
